# Development and validation of a glycolysis-associated gene signature for predicting the prognosis, immune landscape, and drug sensitivity in bladder cancer

**DOI:** 10.3389/fimmu.2024.1430583

**Published:** 2025-01-10

**Authors:** Chong Shen, Yong Suo, Jian Guo, Wei Su, Zhe Zhang, Shaobo Yang, Zhouliang Wu, Zhenqian Fan, Xiaoliang Zhou, Hailong Hu

**Affiliations:** ^1^ Department of Urology, The Second Hospital of Tianjin Medical University, Tianjin, China; ^2^ Tianjin Key Laboratory of Urology, Tianjin Institute of Urology, The Second Hospital of Tianjin Medical University, Tianjin, China; ^3^ Department of Urology, Affiliated Hospital of Hebei University, Baoding, Hebei, China; ^4^ Department of Urology, The Characteristic Medical Center of Chinese People’s Armed Police Force, Tianjin, China; ^5^ Department of Endocrinology, The Second Hospital of Tianjin Medical University, Tianjin, China

**Keywords:** bladder cancer, immune status, glycolysis, gene signature, prognosis

## Abstract

**Background:**

Bladder cancer (BCa) is one of the most common malignancies worldwide, and its prognostication and treatment remains challenging. The fast growth of various cancer cells requires reprogramming of its energy metabolism using aerobic glycolysis as a major energy source. However, the prognostic and therapeutic value of glycolysis-related genes in BCa remains to be determined.

**Methods:**

The fused merge dateset from TCGA, GSE13507 and GSE31684 were used for the analysis of glycolysis-related genes expression or subtyping; and corresponding clinical data of these BCa patients were also collected. In the merge cohort, we constructed a 18 multigene signature using the least absolute shrinkage and selection operator (LASSO) Cox regression model. The four external cohorts (i.e., IMvigor210, GSE32894, GSE48276 and GSE48075) of BCa patients were used to validate the accuracy. We evaluated immune infiltration using seven published algorithms: CIBERSORT, QUANTISEQ, XCELL, TIMER, CIBERSORT-ABS, EPIC, and MCPCOUNTER. Subsequently, in order to analyze the correlation between risk groups(scores) and overall survival, recognised immunoregolatory cells or common chemotherapeutic agents, clinicopathological data and immune checkpoint-related genes of BCa patients, Wilcox rank test, chi-square test, cox regression and spearman's correlation were performed.

**Results:**

Conspicuously, we could see that CD8+ T, cancer associated fibroblast, macrophage M2, NK, endothelial cells and so on were significantly dysregulated between the two risk groups. In addition, compared with the low-risk group, high-risk group predicted poor prognosis and relatively weak sensitivity of chemotherapy. Additionally, we also found that the expression level of partial genes in the model was significantly correlated with objective responses to anti-PD-1 or anti-PD-L1 treatment in the IMvigor210, GSE111636, GSE176307, GSE78220 or GSE67501 cohort; and its expression level was also varied in different objective response cases receiving tislelizumab combined with low-dose nab-paclitaxel therapy based on our mRNA sequencing (TRUCE-01). According to “GSEA” algorithm of R package “clusterProfiler”, the most significantly enriched HALLMARK, KEGG pathway and GO term was separately the ‘Epithelial Mesenchymal Transition’, ‘Ecm Receptor Interaction’ and ‘MF_Extracellular_matrix_structural_constitunet’ in the high- vs. low-risk group. Subsequently, we verified the protein and mRNA expression of interested model-related genes from the Human Protein Atlas (HPA) and 10 paired BCa tissues collected by us. Furthermore, *in vitro* functional experiments demonstrated that FASN was a functional oncogene in BCa cells through promoting cell proliferation, migration, and invasion abilities.

**Conclusion:**

In summary, the glycolysis-associated gene signature established by us exhibited a high predictive performance for the prognosis, immunotherapeutic responsiveness, and chemotherapeutic sensitivity of BCa. And, The model also might function as a chemotherapy and immune checkpoint inhibitor (ICI) treatment guidance.

## Introduction

The most frequent cancer of the urinary system and the tenth most prevalent disease worldwide is bladder cancer (BCa). Because BCa is a diverse illness, there are two clinical subtypes that may be distinguished according to whether it invades the muscle layer: 75% of instances are non-muscle invasive BCa (NMIBC), versus 25% of cases that are muscle invasive BCa (MIBC) (1). In fact, up to 20%–25% of patients with MIBC are diagnosed at the time of their first diagnosis. The majority of bladder cancer deaths are caused by MIBC, and it has a poor long-term survival rate and a high risk of distant metastases (2). BCa histopathology often determines the prognosis in clinical practice (3). It is important to note that each individual differs from the other. The outcome of patients with similar histopathology may also vary.

Energy is provided to the body by glycolysis. Cancer may be indicated by changes in glycolysis. A primary characteristic of cancer cells is an increase in glycolysis, which causes them to convert glucose into lactic acid regardless of oxygen availability. This process is known as the “Warburg effect” (4, 5). A number of malignancies, such as bladder cancer, have an increased affinity for glucose and exhibit a shift toward an aerobic glycolysis-dependent metabolism (6). Increased or abnormal glycolysis has been demonstrated to promote a variety of malignant progressions, as shown in breast, liver, colorectal cancers, etc (7–10). For example, Knibbs et al. (7) found that the long noncoding RNA NEAT1 is upregulated in breast cancer patients, where it directly binds to and forms a scaffold bridge for PGK1/PGAM1/ENO1 complex assembly, thereby enabling highly efficient glycolysis to promote tumor initiation, growth, and metastasis. Li et al. (8) demonstrate that the transcription factor SIX1 promotes aerobic glycolysis in cancer by binding to promoters and recruiting HBO1 and AIB1 to activate glycolytic genes. SIX1 is inhibited by miR-548a-3p, and altering this pathway impacts tumor metabolism and growth. A previous study revealed that HBx triggered aerobic glycolysis through NF-κBp65/HK2 signaling in spontaneous liver cancer, and the excess lactate significantly enhanced HCC cell proliferation via the PI3K/Akt pathway (9). In colorectal cancer, Hong et al. (10) reported that gut F. nucleatum enhanced lncRNA ENO1-IT1 transcription by increasing SP1 binding to its promoter, promoting glycolysis and cancer development. Researches have shown that excessive lactate from abnormal glycolysis encourages tumor growth and invasion, potentially weakening immune defenses and aiding tumor recurrence (11, 12). Thus, targeting glycolysis could be an effective cancer treatment strategy. In addition, there is evidence that increased Warburg effects in BCa contribute to increased tumor aggressiveness and faster proliferation (13).

A crucial component of the TME is the immune infiltrates, which are crucial to the progression of bladder cancer (14). The immune microenvironment can utilize glycolysis to modulate tumor immune response and promote progression (15). A higher level of glycolysis suppress immune cell infiltration, inhibiting antitumor activity (16, 17); thus, it might also contribute to distant metastatic and immune escape (18). It has been demonstrated that glycolytic activity was a prospective predictor of immune signatures in multiple cancers (19). Furthermore, dysregulated glycolysis is also strongly related with radio- and chemo-resistance in cancer (20, 21), manifesting that the inhibition of glycolysis may be an effective strategy to explore optimal combination regimens for the treatment of cancer. Although there have been some previous significant achievements regarding glycolysis in BCa; however, the expression profiles biological functions and mechanisms of BCa-specific glycolysis, especially in the regulation of the tumor microenvironment, has not been completely understood.

In this study, we integrated TCGA_BCa and two microarray datasets (i.e., merge cohort; 664 BCa samples) to compensate for the low sample sizes and to provide more convincing results. This was followed by differential expression analysis between carcinoma and para-carcinoma tissues, survival analysis, co-expression network analysis to determine 34 vital glycolysis-associated gene effecting the prognosis of patients with BCa. We categorized these merge samples as 6 subtypes based on above filtered 34 genes expression profiles by a consensus clustering algorithm. To determine the underlying molecular mechanism of the difference in the prognosis of these subtypes, we further performed differential gene expression analysis between the 6 subtypes and constructed an 18-genes signature using lasso-penalized cox regression analysis; later, the model was validated in multiple external datasets (i.e., IMvigor210, GSE32894, GSE48276 and GSE48075). Surprisingly, the glycolysis-associated gene model could accurately indicate the clinicopathological characteristics and the survival prognosis of patients with BCa. Next, we confirmed the protein and mRNA expression of interested model-associated genes from the Human Protein Atlas (HPA) and 20 paired BCa tissues collected by us. Moreover, the results showed that this signature might participate in the regulation of immune cell infiltration, immune responses and immune checkpoints. Meanwhile, we also further conducted to evaluate the potential role of modeled genes as a biomarker for immunotherapy efficacy in different immunotherapy cohorts (i.e., IMvigor210, GSE111636, GSE176307, GSE78220, GSE67501, and Truce01). Notely, we performed a series of cellular function experiments following FASN knockdown. Collectively, this study systematically analyzed the clinicopathologic features correlation, prognostic value, effects on the immune microenvironment and the underlying molecular mechanism of glycolysis-related gene signature in BCa, which provide a novel orientation to the potential targets.

## Materials and methods

### Extraction of glycolysis-related genes, and data collection and processing

From the MSig database of the Broad Institute (http://www.broad.mit.edu/gsea/msigdb/index.jsp), *a priori* defined glycolysis-related gene set, including 311 genes, were downloaded. A transcriptome and clinical data were downloaded from the TCGA (http://tcga-data.nci.nih.gov/tcga/) and two GEO cohorts (GSE13507, GSE31684) (http://www.ncbi.nlm.nih.gov/geo) for BCa. The TCGA cohort contained 414 tumor tissues and 19 adjacent normal tissues. The two GEO cohort in total contained 250 tumor tissues. Due to a lack of sample size for each cohort, we integrated TCGA cohort and two GEO cohort to get merge cohort (13449 genes, 662 tumor samples). Next, 88 glycolysis-related genes with prognostic values were screened from 311 glycolysis-related genes by univariate Cox regression analysis of overall survival (OS), based on the TCGA dataset. In the TCGA cohort, using the “limma” R package, 128 differentially expressed glycolysis genes were identified between tumor tissues and adjacent normal tissues with a false discovery rate (FDR)< 0.05. Therefore, we identified 34 glycolysis-related DEGs associated with prognosis by intersecting 88 prognostic glycolysis-related genes with 128 differentially expressed ones. The Kaplan-Meier curve was used to determine whether the high or low expression of each gene of these 34 genes was correlated with the OS in patients with BCa (p<0.05). Moreover, an analysis and visualization of these 34 gene mutations was also conducted using the “maftools” R package.

The IMvigor210 cohort (obtained from the R package “Imvigor210coreBiologies”), an 348 urothelial carcinoma cohort treated with the anti-PD-L1 antibody atezolizumab, was used for validation of glycolysis-associated gene signature. The microarray expression data of other three GEO validation datasets, GSE48276 (n = 116), GSE48075 (n = 73), and GSE32894 (n = 224), were all quantile-normalized, and the genes were annotated in their separate microarray platform files GPL14951, GPL6947, and GPL6947. Additionally, the microarray expression data of three GEO immunotherapy datasets, GSE111636 (n = 11), GSE176307 (n = 89), GSE78220 (n = 28), and GSE67501 (n = 11) were all quantile-normalized, and the genes were annotated in their respective microarray platform files GPL17586, GPL24014, GPL11154, and GPL14951. Notably, in our single-arm phase 2 trial (term_id, TRUCE-01; registration number, NCT04730219), tislelizumab (200mg) combined with low-dose nab-paclitaxel (200mg) also preliminary confirmed clinical benefits and safety in the therapy of muscle-invasive urothelial bladder carcinoma patients (22). Therein, tislelizumab is a novel humanized monoclonal antibody programmed death receptor-1 (PD-1) inhibitor and shows a predictable and manageable safety/tolerability profile in patients with PD-L1^+^ UC (23). The clinical traits information, survival data or the immunotherapeutic efficacy of above these datasets are shown in **Supplementary File S1** and **Supplementary File S2**, respectively.

### Identification of bladder cancer subtypes, and a series of association analysis

Based on the 34 genes’ expression, the “ConsensusClusterPlus” R package was utilized to identify the possible molecular subtypes of bladder cancer in the merge cohort (24). Clustering was performed using the k-means method and similarity distance calculated according to Euclidean distance. A clustering algorithm of 1000 iterations was used, with 80% of the samples in each iteration, and a CDF curve of the consensus score was used to calculate the optimal cluster number. The glycolysis-related 34 DEGs associated with prognosis were selected to perform principal component analysis (PCA) analysis. Afterwards, the Kaplan–Meier (KM) method and log-rank test were used to compare overall survival (OS), disease-free survival (DFS), disease-specific survival (DSS), and progression-free survival (PFS) among subtypes of BCa patients. Next, Correlations between subtype groups and clinicopathological data, recognized immunoregolatory cells of BCa patients were performed using chi-square test and wilcox rank test.

GSVA is used for evaluating KEGG gene set enrichment using the “GSVA” R package, and significantly differential pathways between molecular typing were selected using the adjusted p-value<0.05 criterion. Single-sample gene set enrichment analysis (ssGSEA) was used to quantify the enrichment score of 29 immune signatures, and association analysis with subtypes.

### Model construction of the glycolysis-associated gene signature and validation

As a first step, we identified 3754 differentially expressed genes among the 6 clusters (|log2FoldChange| >1, false discovery rate (FDR) <0.05). Using the “survival” R package, we performed univariate Cox regression analyses of the DEGs, and got 1223 genes with prognostic values. Following that, LASSO analysis using the R package “glmnet” and multivariate Cox regression analysis using the “survival” R package were performed to establish a risk model. Risk scores were calculated using the “predict” function in R software, and risk scores are calculated according to the following mathematical model: Risk score = h_0_ (t) ∗ exp(β_1_ X _1_ + β_2_ X _2_ + … + β_n_ X _n_); this equation is expressed as follows: n = the number of model genes to be modeled; β= correlation coefficient and X = expression level of each model gene predicted; and h_0_ (t) originated from the”predict” function.

Using the median risk score as a cutoff value, 664 BCa patients in the training merge cohort were divided into high- and low-risk groups. Kaplan-Meier (KM) survival and time-dependent receiver operational feature (ROC) and calibration curves were plotted by “survival”, “timeROC” and “RMS” R packages to assess the discrimination and calibration of the glycolysis-related 18-gene model. And, risk score distribution, risk status, and risk heat map were utilized for assessing the risk of predictive models. Besides that, the glycolysis-associated 18-gene model was validated in four independent test cohort (i.e., IMvigor210, GSE48276, GSE48075, and GSE32894). Clinicopathological and survival information of these datasets was obtained from the TCGA and GEO data portal and manually collected. Moreover, the partial modeled genes expression in the signature was further validated through the Human Protein Atlas database (HPA) and real-time quantitative PCR (qRT-PCR).

### The associations between model and clinical features; independent predictor; predictive nomogram construction

Wilcox test or chi-square test was applied to investigate the clinical correlations of the modeled risk scores/group and multiple clinical traits. The Spearman rank correlation analysis was carried out in analyzing the associations between riskscores and clinical factors of BCa patients. To identify independent factors for OS in BCa patients, we applied univariate and multivariate cox regression analysis for the gene signature and other clinicopathological features (gender, age, T/N status and grade). Additionally, the prognostic nomogram and corresponding calibration plots was produced by the “RMS” R package.

### Correlation analyses between glycolysis-related gene model and immune checkpoint, immune cell infiltration, tumor mutation burden and immunophenoscore

To explore the efficacy of treatment response, we employed the expression of immune checkpoint between high- and low-risk groups. Subsequently, to assess the immune-infiltration in BCa, we conducted the 7 immune-infiltration algorithm (TIMER, CIBERSORT, CIBERSORT-ABS, QUANTISEQ, MCPCOUNTER, XCELL and EPIC) to calculate the proportion of various immune cells and revealed the function of immune-infiltration via multiple strategies. The Wilcox rank-sum test were applied to analyze the differences between the two riskscore groups; then, the results were visualized by heatmaps through the R package “Pheatmap”. Associations between riskscore and immune-infiltration cells were analyzed by the Spearman rank correlation analysis. And, the single-sample gene set enrichment analysis (ssGSEA) algorithm was also adopted to evaluate the score level of 29 immune infiltration/function between high- and low-risk groups. TMB and IPS are all superior predictor of the response to immune checkpoint blockades (ICBs; anti-PD1, or anti-CTLA4, etc.), which quantifies the determinants of tumor immunogenicity and characterizes the intratumoral immune landscapes. Hence, we could predict the immunotherapy response of each BCa patient using our model.

### Gene set enrichment analysis and drug sensitivity analysis

To further understand and identify enriched cellular pathways associated with the model in BCa, R package clusterProfiler was used for GSEA to compare the different Hallmark, KEGG pathways or GO terms download from MSigDB (http://software.broadinstitute.org/gsea/msigdb) between the two risk groups from the merge data. Then, to explore the connection between glycolysis-related gene expression levels and drug susceptibility, we downloaded the drug sensitivity information from the CellMiner database and used the National Cancer Institute (NCI)-60 analysis tool to analysis. Next, the “pRRophetic” package was applied to obtain the half-maximal inhibitory concentration (IC50) of two riskscore groups to Cisplatin, Docetaxel, Paclitaxel and Vinblastine.

### RNA extraction and quantitative real-time PCR

In this study, 10 pairs of BCa tissues and adjacent normal tissues were obtained from the urology department of Tianjin Medical University’s Second Hospital. Patients were required to complete a permission form before utilizing therapeutic resources. Total RNA Kit (Omega, Norcross, USA) was performed to obtain the total RNA from BCa tissues and adjacent normal tissues. Next, 3g of total RNA was converted to cDNA using the RevertAid First Strand cDNA SynthesisKit (Thermo Fisher Scientific, Waltham, USA). qRT-PCR test utilized TOROGreen qPCR Master Mix (Toroivd, Shanghai, China) on an ABI 7900HT rapid real-time PCR equipment (Applied Biosystems, Waltham, USA) to measure mRNA expression levels. GAPDH was used as an internal control. The relative quantitative value of target gene was determined by 2^−ΔΔCT^ method. The primer sequences are displayed in **Table 1**.

**Table 1:** The primers used for real-time PCR are designed and synthesized by Sango Biotech (Shanghai, China).

Gene Name	Primer Type	Primer Sequence	Product Length
SPINK4	Forward primer	5’-CAGTGGGTAATCGCCCTGG-3’	100
	Reverse primer	5’-CACAGATGGGCATTCTTGAGAAA-3’	
DMRTA1	Forward primer	5’-GCAGAGACCGAGGCGTTAG-3’	107
	Reverse primer	5’-AACCTGCATCCCCGATGGTA-3’	
SPINK5	Forward primer	5’-TGCTTTTCGGCCCTTTGTTAG-3’	107
	Reverse primer	5’-CACACATTGCACACTTATTGCC-3’	
SLC1A6	Forward primer	5’-TGCGCCCATATCAGCTCAC-3’	99
	Reverse primer	5’-CAATGAGAGGTAACACCAGCAT-3’	
FASN	Forward primer	5’-CCGAGACACTCGTGGGCTA-3’	209
	Reverse primer	5’-CTTCAGCAGGACATTGATGCC-3’	
GAPDH	Forward primer	5’-CGGAGTCAACGGATTTGGTC-3’	180
	Reverse primer	5’-TTCCCGTTCTCAGCCTTGAC-3’	

### Cell transfection

Transfection can be done when cells accounts for 60–80% of container. In a six-well plate, the transfection dose for each well was 100nM SiRNA or negative control RNAi 100-nM with LipofectamineTM 2000 (Invitrogen) according to the manufacturer’s protocol. After 48 h, the cells were collected for western blotting, CCK-8, wound Healing, transwell assays.

### Western blot analysis

Total protein was extracted from T24 and UM-UC-3 cell lines as well as tumor tissues using RIPA buffer (BOSTER) supplemented with PMSF. Protein concentration was determined using a BCA assay kit. Equal amounts of protein samples (25 μg per lane) were separated by SDS-PAGE on a 10% acrylamide gel, then transferred onto a polyvinylidene difluoride (PVDF) membrane (Millipore, Billerica, MA). The membrane was blocked with 5% non-fat milk and incubated overnight at 4°C with the following primary antibodies: rabbit anti-FASN (dilution 1:5,000; proteintech) and mouse anti-β-actin (dilution 1:5,000; proteintech). Subsequently, the PVDF membrane was washed and incubated with anti-rabbit or anti-mouse IgG at room temperature for 1 hour. Chemiluminescence was used to detect immune-reactive bands, and relative intensities were measured and analyzed using ImageJ software.

### Cell proliferation assays

Proliferation was detected with CCK-8 (Beyotime Institute of Biotechnology). Briefly, 2×10^3^ cells were cultured in 100 µL medium in 96-well plates, incubated with 10 µL reagent for 2 h, and analyzed using a microplate reader at a wavelength of 450 nm. For the colony formation assay, 1 × 10^3^ cells were seeded in a 6-well plate and cultured for one week, followed by fixing with 4% paraformaldehyde and staining with 0.25% crystal violet.

### Cell invasion assays

For the cell invasion assays, 8 μm micropore inserts in 24-well cell culture plates were used. For cell invasion experiments, 5 × 10^4^ cells were seeded into upper wells coated with 50 μL diluted matrigel without FBS. In transwell assay, 10% FBS was added to lower wells. Wells were fixed with 4% paraformaldehyde for 30 min and stained with 0.25% crystal violet for 20 min.

### Wound healing assay

T24 and UM-UC-3 cells were seeded on 6-well plates and grown to ply overnight. Twenty-four hours after transfection, plot the channel on the monolayer of cells with a 10 μL micropipette tip. T24 and UM-UC-3 cells were then rinsed with PBS and incubated for an additional 24 hours in serum-free medium at 5% CO2, 37°C.

### Statistical analysis

All analyses were completed by using R programming language (version 4.1.2) and its relevant packages, as well as Graphpad Prism 8.0.2. Wilcox’s test compared variables between two risk score groups. Chi-square tests examined the link between risk groups and clinicopathological features. Spearman’s correlation was employed to assess group correlations. The Kaplan-Meier curve analyzed survival data. The R package time performed the ROC analysis. Univariate and multivariate Cox regression analyses identified independent prognostic factors. A two-sided P < 0.05 was considered statistically significant. Furthermore, p-value summaries were as follows: ****, P < 0.0001; ***, 0.0001 < P < 0.001; **, 0.001 < P < 0.01; *, 0.01 < P ≤ 0.05.

## Results

### Identification of glycolysis-related DEGs associated with prognosis, and map of these genes variants across the fused merge dataset

**Figure 1:**
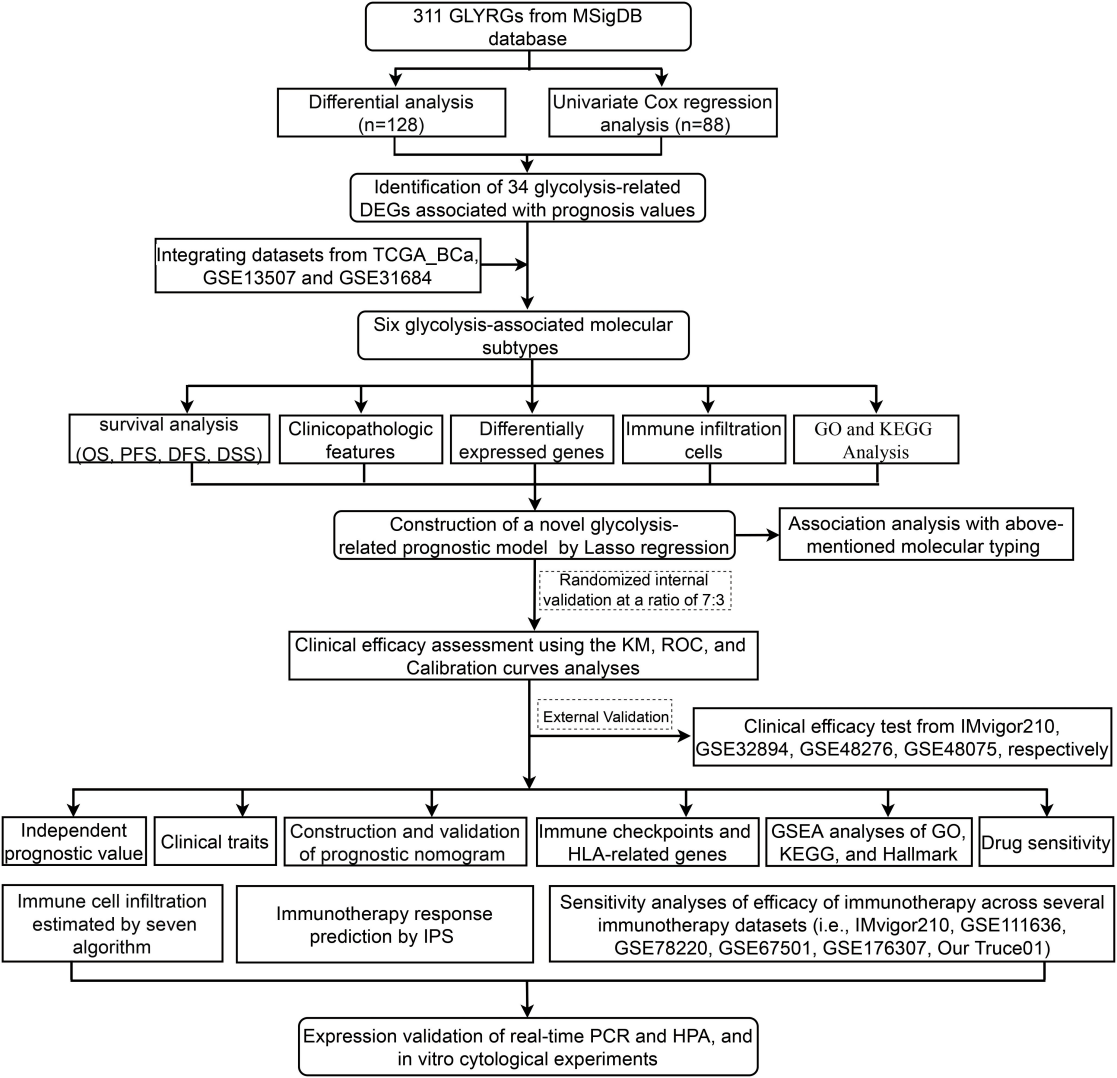
Flow diagram of the study.

A flow diagram for the present study can be found in **Figure 1**. The 311 glycolysis-related genes were extracted from GSEA database. After unicox analysis, we got 88 glycolysis-related genes which could affect prognosis. The expression of 128 glycolysis-related genes were differential between bladder cancer samples and adjacent normal tissues in TCGA database. When the 88 prognosis-related genes and the 128 differentially expressed genes overlapped, a total of 39 genes was obtained (**Supplementary Figure S1A**). As shown in the heatmap, most of the 39 overlapping genes showed an upregulation in tumor tissue (**Supplementary Figure S1B**). It can be seen from the forest plots using the univariate cox analysis that most of the genes are high-risk genes (**Supplementary Figure S1C**). STRING’s PPI network showed the candidate genes’ interactions. The interaction network among these genes indicated that G6PD, GAPDH and PFKM were the hub genes (**Supplementary Figure S1D**). In order to follow-up research and expand the sample size, we integrated TCGA dataset and GEO dataset (GSE13507, GSE31684) to obtain merge dataset (13449 genes, 662 tumor samples). Finally, 34 DEGs derived from the intersection of TCGA-DEGs, GSE13507 and GSE31684 as the research object were ultimately selected. It can be noted that these 34 differentially expressed genes exhibit a higher prognostic value, based on both univariate Cox regression analysis and Kaplan Meier (KM) curves (**Table 2**). These specific 34 genes status of CNVs and mutations are also depicted in **Supplementary Figure S2**. Among these, copy number amplifications (>10%) were encountered in 5 genes (B3GAT3, SOX9, GALK1, PGM2L1 and PMM2) and copy number losses (>10%) in 2 genes (CHPF and GMPPA) (**Supplementary Figure S2A**). Both chromosome 12 and 20 had the highest number of five genes (**Supplementary Figure S2B**). Moreover, mutation of each gene in different samples is shown in waterfall plot (**Supplementary Figure S2C**). Of these, MUP205 has the highest frequencies of gene mutation (6%); however, the remaining genes were less than or equal to 1%.

**Table 2:** According to TCGA-2GEO merge cohort, both uniCox regression and KM curves revealed 34 glycolysis-related genes with significantly prognostic significance.

Gene ID	HR(uniCox)	HR.95L (uniCox)	HR.95H (uniCox)	Pvalue (uniCox)	Pvalue (KM)
PYGB	1.177124973	1.044121603	1.327070716	0.00768163	0.001749207
ME1	1.084205115	0.993924492	1.182686151	0.068366637	0.004008069
PGM2L1	1.151544067	1.031316165	1.285787796	0.012139699	0.001545547
PPP2CB	1.28290605	1.082445567	1.520490252	0.004054996	0.000563397
CHPF	1.297704898	1.141294582	1.475550683	6.98E-05	2.45E-05
NUP205	1.21760277	1.036996391	1.429664094	0.016241653	0.000619682
TPST1	1.193933679	1.080990067	1.318677824	0.00047244	1.27E-06
CASP6	0.813637296	0.690735563	0.958406783	0.013570739	0.000947284
RBCK1	0.901322173	0.766342621	1.060076312	0.209428077	0.026685019
AURKA	1.168342073	1.058328553	1.289791526	0.00204581	8.57E-05
IDUA	0.89027279	0.804555799	0.985122028	0.024438389	0.00230427
PLOD1	1.375190984	1.200110929	1.575812865	4.53E-06	4.79E-06
SOX9	1.074959198	1.013340943	1.140324277	0.016395396	0.000311498
SLC2A3	1.179306971	1.096667766	1.268173439	8.61E-06	7.66E-06
CHST6	1.236625803	1.070149196	1.429000164	0.003989403	8.96E-05
B3GALT6	1.239814106	1.048899916	1.465477299	0.01175075	0.006558229
RAE1	1.293511489	1.056870863	1.583137571	0.012541781	0.003723595
PAM	1.131902109	1.016094115	1.260909168	0.024455926	0.001989656
TPI1	1.249796363	1.072907205	1.455849064	0.004186498	0.000871341
B3GNT3	0.955375463	0.891375205	1.023970905	0.196916662	0.04246574
B3GAT3	1.267888396	1.048140084	1.533708147	0.014520599	0.001957934
PMM2	1.178801633	1.009281304	1.376794839	0.037839546	0.001627931
ALDH1A3	1.082720142	1.009410103	1.161354441	0.026297025	0.004081642
GAPDH	1.18299621	0.998451236	1.401650859	0.052130802	0.000446617
STC1	1.150893854	1.055898025	1.25443616	0.00138651	0.001464813
SPAG4	0.918216705	0.839236999	1.004629108	0.062981795	0.003126145
HSPA5	1.203387875	1.018013956	1.422517215	0.030072056	0.006643677
G6PD	1.174422841	1.060490716	1.300595082	0.002014981	0.001332806
ENO1	1.363641011	1.170651508	1.588446088	6.79E-05	0.000136222
DCN	1.087284071	1.023407483	1.155147554	0.006749106	0.000448517
GMPPA	1.378955387	1.124226987	1.691400387	0.002044367	0.000475416
KDELR3	1.162056678	1.064221595	1.268885851	0.000816627	8.91E-05
PFKM	1.066270934	0.92795224	1.225207134	0.365377832	0.075171376
GALK1	1.491099325	1.276840983	1.741310961	4.47E-07	1.75E-07

### Identification of molecular subtyping and its prognostic significance based on the above merge BCa dateset

**Figure 2:**
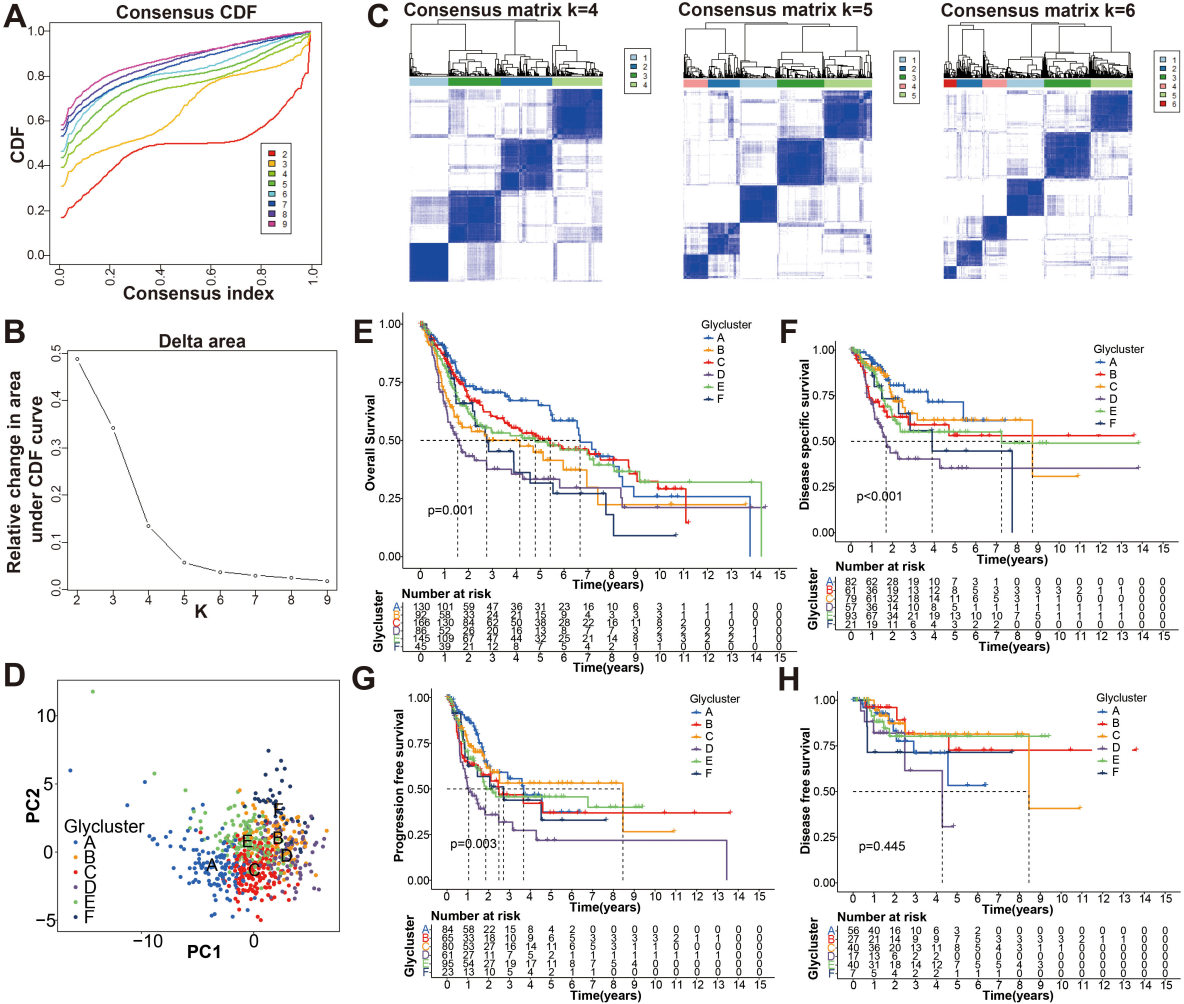
Identification the glycolysis-associated molecular subtypes of BCa. **(A)** Cumulative distribution function (CDF) curves of the consensus scores for different subtype numbers (k = 2-9). **(B)** CDF delta area curve with k = 2 to 10. **(C)** Heatmaps of consensus matrices for k = 4, 5, 6 based on CDF curve estimation. **(D)** Principal component analysis (PCA) for six molecular typing. A single point represents each sample, and each subtype is represented by a different color. **(E-H)** KM survival analysis of OS, DSS, PFS, and DFS for the six subtypes.

First, the expression pattern of the 34 genes was used to probe the possible bladder cancer clusters from the merge cohort. All bladder samples were divided into k (k = 2-9) clusters using “Consensus Cluster Plus” R package. According to the CDF and CDF delta area curves of the consensus score, and heatmaps of consensus matrices, the most stable cluster when k = 6 was delineated (**Figures 2A–C**). Thus, six molecular subtypes were established. To test this further, we carried out PCA analysis (**Figure 2D**). Moreover, Kaplan-Meier curves showed that the 6 clusters differ significantly in terms of overall survival rates (OS), disease specific survival rate (DSS) and progression free survival rate (PFS) (p < 0.01), whereas not disease-free survival (DFS) (**Figures 2E-H**).

**Figure 3:**
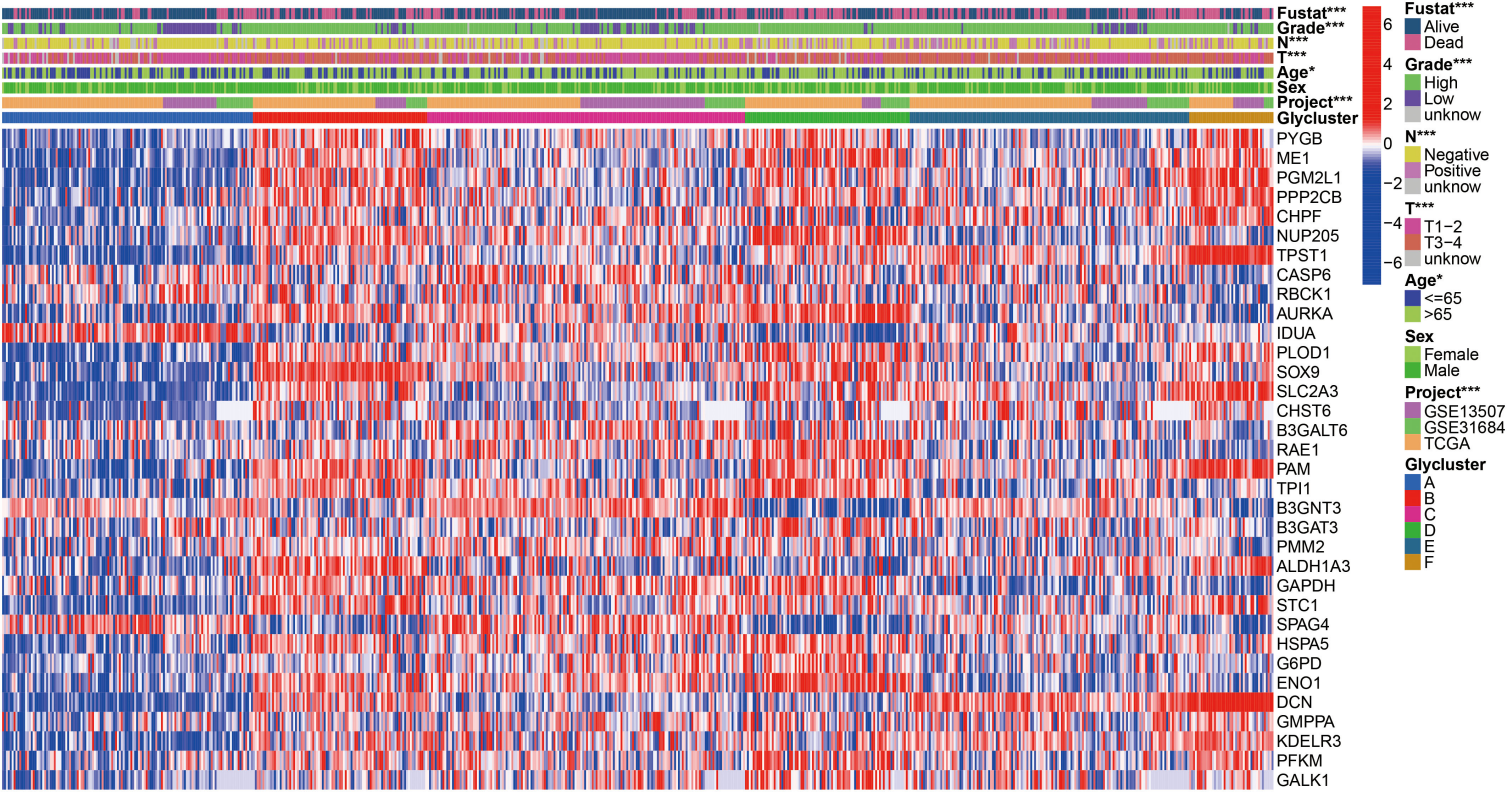
Heatmap of 34 glycolysis-related prognosis DEGs, and the chi-square analysis between corresponding the six molecular subtypes and commonly clinical traits. *p < 0.05, ***p < 0.001.

To further search the significance of several clinical traits (fustat, age, sex, T stage, N stage and grade) in the six clusters, chi-square test was carried out. As shown on the tip of the heatmap, the clinical traits (fustat, age, T stage, N stage, grade) showed a significant distribution in the 6 clusters (p <0.05), whereas sex has no dramatic difference (p > 0.05) (**Figure 3**). Heatmap also demonstrated the expressional profiles of the 34 glycolysis-associated prognosis genes at six molecular subtypes in merge cohort (**Figure 3**). We can see cluster A has the best OS, meanwhile cluster D has the worst OS. To explore the underlying molecular mechanism of above 6 molecular typing, we next performed GSVA of KEGG pathway gene sets to figure out dynamics of biological processes and pathways between every two typing based on merge dataset (**Supplementary Figure S3**; **Supplementary File S3**). With ssGSEA algorithms, we further explored the interrelation between the 6 clusters and immune status. The infiltration levels of CD56dim-natural-killer-cellna, Monocytena and Type17-T-helper-cellna were obviously higher in cluster A versus the other subtypes. Cluster B were characterized by a obviously higher infiltration level of Type2-T-helper-cellna, Neutrophilna, Activated-CD8-T-cellna and Activated-CD4-T-cellna (**Supplementary Figure S4**).

### Construction and internal validation of the glycolysis-associated gene signature based on merge cohort

In order to uncover the hidden cause of the differences in clinical and immune characteristics between the six clusters in the merge cohort, we compared the differences in mRNA expression of the 6 clusters, and identified 3754 genes that were differentially expressed between the clusters. (FDR <0.05, |log2FoldChange| >1) (**Supplementary Figure S5**; **Supplementary File S4**). To better understand the functional pathways involved in the 3754 DE-genes in BCa, GO function and KEGG pathway enrichment analyses were performed. We got 883 enriched GO terms (777 BP, 63 CC and 43 MF) and 37 KEGG pathways with adjusted p-value <0.0001, which were presented at **Supplementary Figure S6** and **Supplementary File S5**. Univariate Cox regression analysis was used to analyze the above 3754 differentially expressed genes to determine their clinical value and obtained 1223 genes (P <0.01) (**Supplementary File S6**). After that, LASSO regression was used to remove the genes that show strong collinearity. The independent prognosis-related genes were validated with a multivariate Cox regression analysis (**Supplementary Figure S7**). Finally, we got 18 of the 1223 genes for risk scoring model: CPNE8, CXCL6, COMP, SPINK4, HLA−DQB2, CLIC3, DMRTA1, MAP2, ZNF600, CYTL1, DIP2C, FOXC2, LPXN, SPINK5, SLC1A6, FASN, SCD and EGFL6. As showing in the boxplot, we could see the correlation between the model genes and the six clusters (**Supplementary Figure S8**). For example, SPINK4 and ZNF600 are markedly expressed in subtype A, while MAP2 is markedly expressed in subtype D, which means that they may be marker genes for the two subtypes, respectively.

**Figure 4:**
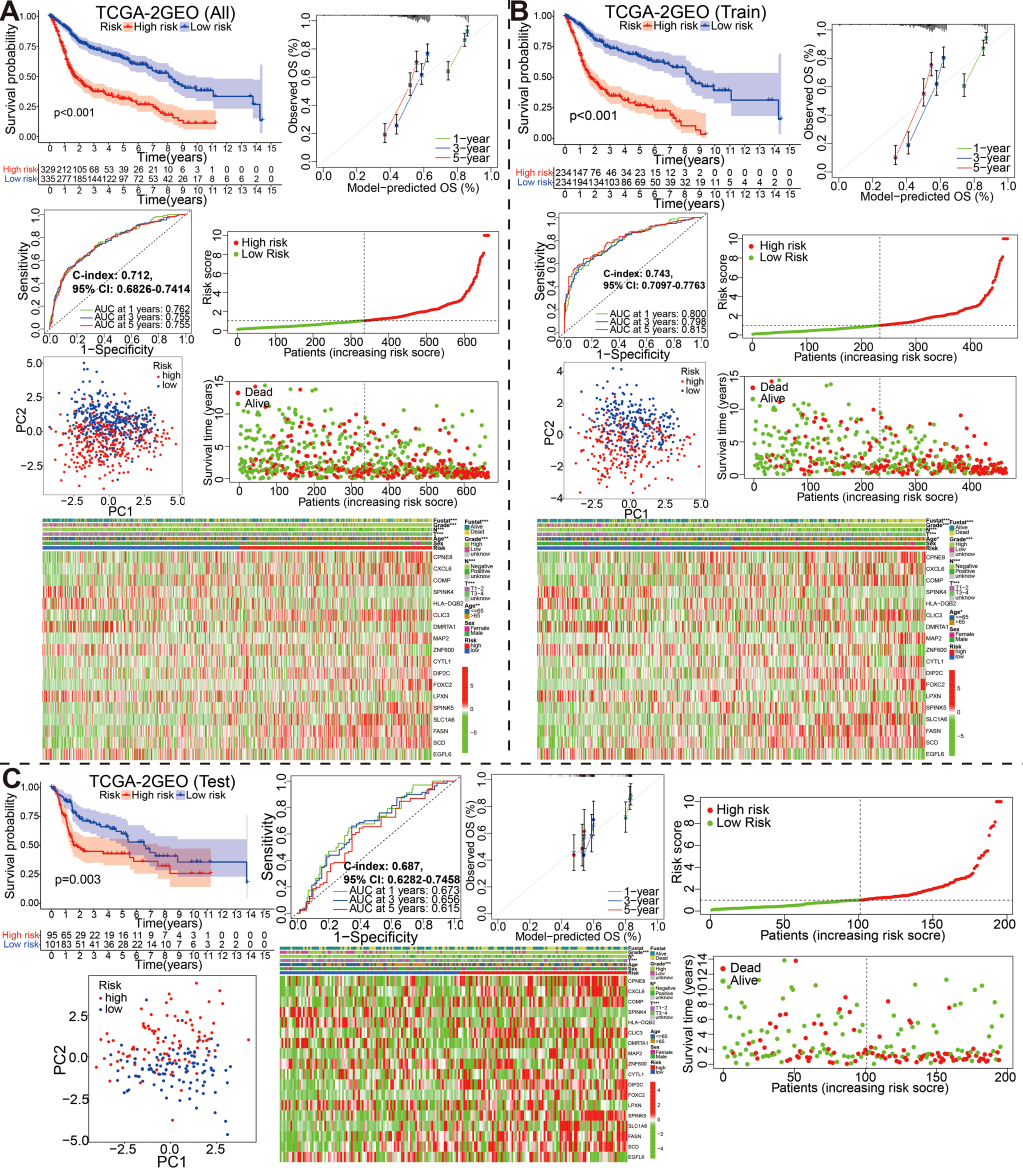
Stratification of overall, training and self-validation set from the fused merge dateset. Kaplan-Meier survival plots, ROC curves for 1-, 3-, 5-years along with the c-index, calibration curve, the distribution of risk score ranking and survival status, model gene expression heatmaps with order of increasing risk score, and PCA analyses in the all **(A)**, training **(B)** and self-validation set **(C)**.

Subsequently, we stochastically divided the 662 bladder cancer patients from merge cohort into training set and self-validation set at a ratio of 7:3 (**Table 3**). A median risk score was used to categorize all patients into high risk or low risk groups. Kaplan-Meier survival curve, the ROC analysis and the calibration curves of 1-, 3-, and 5-year OS from overall (**Figure 4A**), training (**Figure 4B**) and self-validation set (**Figure 4C**) were plotted to assess accuracy of the prognostic model. As revealed by Kaplan-Meier survival analysis, the OS of patients with BCa at high risk was significantly lower than that of patients at low risk, among the all, training and self-validation sets (P < 0.001, P < 0.001 and P = 0.003). An analysis of ROC curves showed good predictive performance for the overall (1-year AUC =0.762, 3-year AUC = 0.755, 5-year AUC = 0.755), so does the training set and internal self-validation set. Furthermore, the concordance index (C-index) for the overall set, the training set and internal validation set, was separately 0.712 (95% CI: 0.6826-0.7414), 0.743 (95% CI: 0.7097-0.7763), and 0.687 (95% CI: 0.6282-0.7458). Furthermore, the risk score model, including risk score ranking, living status, gene expression heat maps and survival plot were further explored for overall, training and self-validation sets and shown in **Figures 4A–C**, respectively. Patients with high risk group had higher mortality rates than those with low risk group when we compared their survival times. Analysis of heatmaps revealed expression profiles of the 18 modeled genes in high risk and low risk group. Corresponding risk group, age, gender, grade, T, N, and fustat were also shown the top of the heatmap. In addition, we conducted other external validation dataset analysis to evaluate the prognostic model’s effectiveness and accuracy in clinical practice.

**Table 3:** The merge dataset (662) was randomly divided into Train (467) and Test (195) groups.

Covariates	Type	Total cohort (662)	Testing internal cohort (195)	Training cohort (467)	Pvalue*
Sex	Female	162 (24.47%)	48 (24.62%)	114 (24.41%)	1
	Male	500 (75.53%)	147 (75.38%)	353 (75.59%)	
Age	<=65	263 (39.73%)	89 (45.64%)	174 (37.26%)	0.0546
	>65	399 (60.27%)	106 (54.36%)	293 (62.74%)	
T	T1-2	289 (43.66%)	89 (45.64%)	200 (42.83%)	0.7108
	T3-4	340 (51.36%)	99 (50.77%)	241 (51.61%)	
	unknow	33 (4.98%)	7 (3.59%)	26 (5.57%)	
N	Negative	434 (65.56%)	138 (70.77%)	296 (63.38%)	0.2985
	Positive	170 (25.68%)	46 (23.59%)	124 (26.55%)	
	unknow	58 (8.76%)	11 (5.64%)	47 (10.06%)	
Grade	High	528 (79.76%)	152 (77.95%)	376 (80.51%)	0.6471
	Low	131 (19.79%)	41 (21.03%)	90 (19.27%)	
	unknow	3 (0.45%)	2 (1.03%)	1 (0.21%)	
Fustat	Alive	388 (58.61%)	121 (62.05%)	267 (57.17%)	0.2824
	Dead	274 (41.39%)	74 (37.95%)	200 (42.83%)	

*Significant difference (p < 0.05 using Chi-square test).

### The clinical outcome predictability of the signature combined with clinicopathological characteristics

In order to study the relationship between the glycolysis-related gene signature and clinical features, we used chi-square or wilcox nonparametric tests across the fused merge set to compare risk scores between different clinical features (**Supplementary Figure S9**). As presented in **Supplementary Figure S9A**, patients with high grade had higher risk scores. Moreover, T3-4 patients, >65 years patients and N1-3 exhibited higher risk scores (P<0.05). Furthermore, the chi-square test was also performed to assess the association between the risk score group and available clinical parameters, in order to confirm the clinical value of the signature (**Supplementary Figure S9B**). There were significantly correlations between risk score group and age, grade, fustat, T, or N. We also conducted association analysis between the risk score and published TCGA_BCa immunotyping results (**Supplementary Figure S10**). Obviously, patients with immune C1 or C2 than C3 or C4 had higher risk scores.

**Figure 5:**
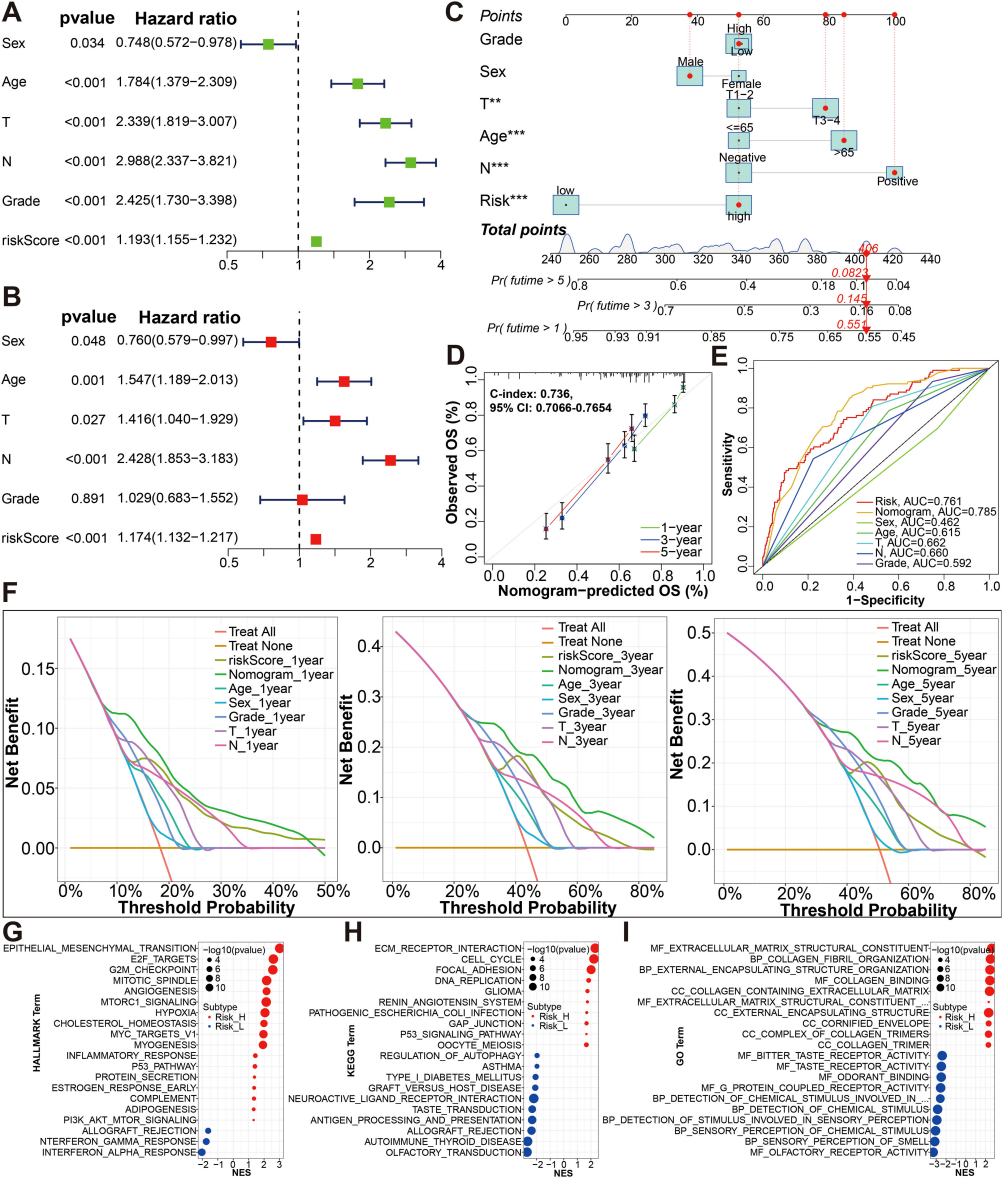
Establishment and evaluation of the nomogram. **(A, B)** The risk score was independent risk factors for BCa by univariate and multivariate Cox regression analyses of overall survival. **(C)** Construction of a glycolysis-related gene signature combined with clinical features nomogram for predicting the 1-, 3- and 5-year OS rates. **(D)** Calibration curve of nomogram. **(E)** A multi-index time-dependent ROC analysis was employed to evaluate the predictive accuracy of the glycolysis-related gene signature or our nomogram, and compare it with other clinical traits. **(F)** DCA of 1-, 3- and 5-year was applied to render clinical validity to the constructed gene signature or nomograms. **(G–I)** The most significant HALLMARK, KEGG pathways and GO functional enrichment in the high risk and low risk groups by GSEA method are displayed. *p < 0.01, ***p < 0.001.

Furthermore, risk score was shown to independently impact OS in the univariate and multivariate Cox proportional hazards models (univariate Cox: HR (95% CI), 1.193 (1.155-1.232), P < 0.001; multivariate Cox: HR (95% CI), 1.174 (1.132-1.217), P<0.001; **Figures 5A, B**). The results of this study suggest that our risk score is also a viable independent prognostic factor for bladder cancer patients. In order to provide a clinically practical tool for predicting the probability of 1-, 3- and 5-year OS, we structured a nomogram using commonly clinic-pathological features (age, sex, grade, T stage, and N stage) and the risk score (**Figure 5C**). According to our constructed nomogram, the calibration line agreed well with the curve calculated (**Figure 5D**). When ROC curves were compared between the nomogram’s prognostic accuracy and clinical features, the nomogram showed greater predictive power and accuracy (**Figure 5E**). The concordance index (C-index) for the nomogram was 0.736 (95% CI: 0.7066-0.7654). There was a greater accuracy in survival predictions at 1-, 3- and 5-years based on the decision curve (**Figure 5F**). To explore the underlying molecular mechanism of the glycolysis-related prognosis model, we next conducted GSEA of HALLMARK, KEGG and GO gene sets to figure out difference of biological function and pathways between the high- and low-risk groups based on the merge cohort (**Figures 5G–I**; **Supplementary File S7**). Of them, the most significantly enriched 20 HALLMARK terms (including, ‘Epithelial Mesenchymal Transition’, ‘E2F Targets’, ‘G2M Checkpoint’, ‘Angiogenesis’ and ‘Hypoxia’ in the high-risk group, etc.) are shown in **Figure 5G**. For KEGG terms, GSEA results from KEGG terms revealed that the high-risk group was primarily enriched in “ECM receptor interaction”, “cell cycle”, “focal adhesion”, “DNA replication”, “GAP junction”, “P53 signaling pathway” (**Figure 5H**). For GO terms, the high-risk group was primarily enriched in “MF-extracellular-matrix-structural-constituent”, “BP_collagen fibril organization”, “BP_external encapsulating structure organization”, “MF_collagen binding” and “CC_collagen containing extracellular matrix”, and the low-risk group was primarily enriched in “MF_bitter taste receptor activity”, “MF_odorant binding”, “MF_G protein coupled receptor activity”, “BP_detection of chemical stimulus”, and “BP_detection of stimulus involved in sensory perception” (**Figure 5I**).

Subsequently, BCa patients were divided into subgroups according to age, stageT, stageN, gender and grade, respectively, and KM analysis was further conducted in each subgroup. We can see high risk group with a bad prognosis in the two age subgroups (P <0.001), T1-2 and T3-4 subgroups (P <0.01), both N subgroups (P <0.01), both gender subgroups (p<0.01), both survival status subgroups (p<0.001), and high grade subgroup (P <0.001); however, high risk group did not show a bad prognosis in the low grade subgroup (P = 0.156) (**Supplementary Figure S11**).

### The external validation of the glycolysis-associated gene signature based on four additional cohorts

**Figure 6:**
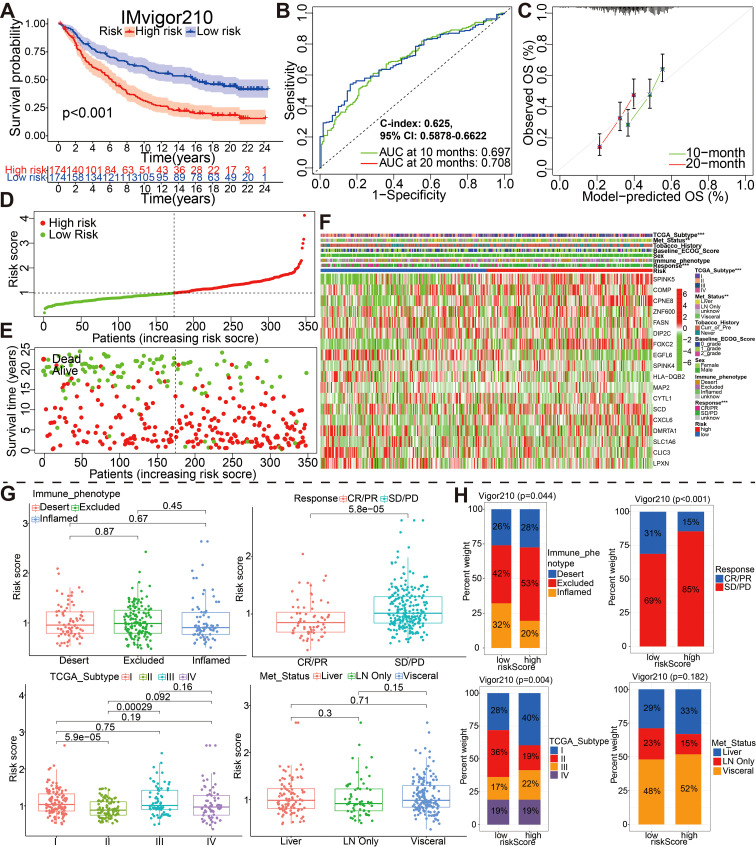
External validation of the 18 model genes signature in the IMvigor210 cohort by different aspects such as **(A)** survival outcomes, **(B)** ROC curves, **(C)** calibration plots, **(D)** the distribution of risk scores, **(E)** the distribution of overall outcomes and **(F)** the gene expression heatmap analysis. **(G, H)** Correlation between riskscore group and clinicopathological data of BCa patients via Wilcox rank test or Chi-square test. CR, complete response; PR, partial response; SD, stable disease; PD, progressive disease.

**Figure 7:**
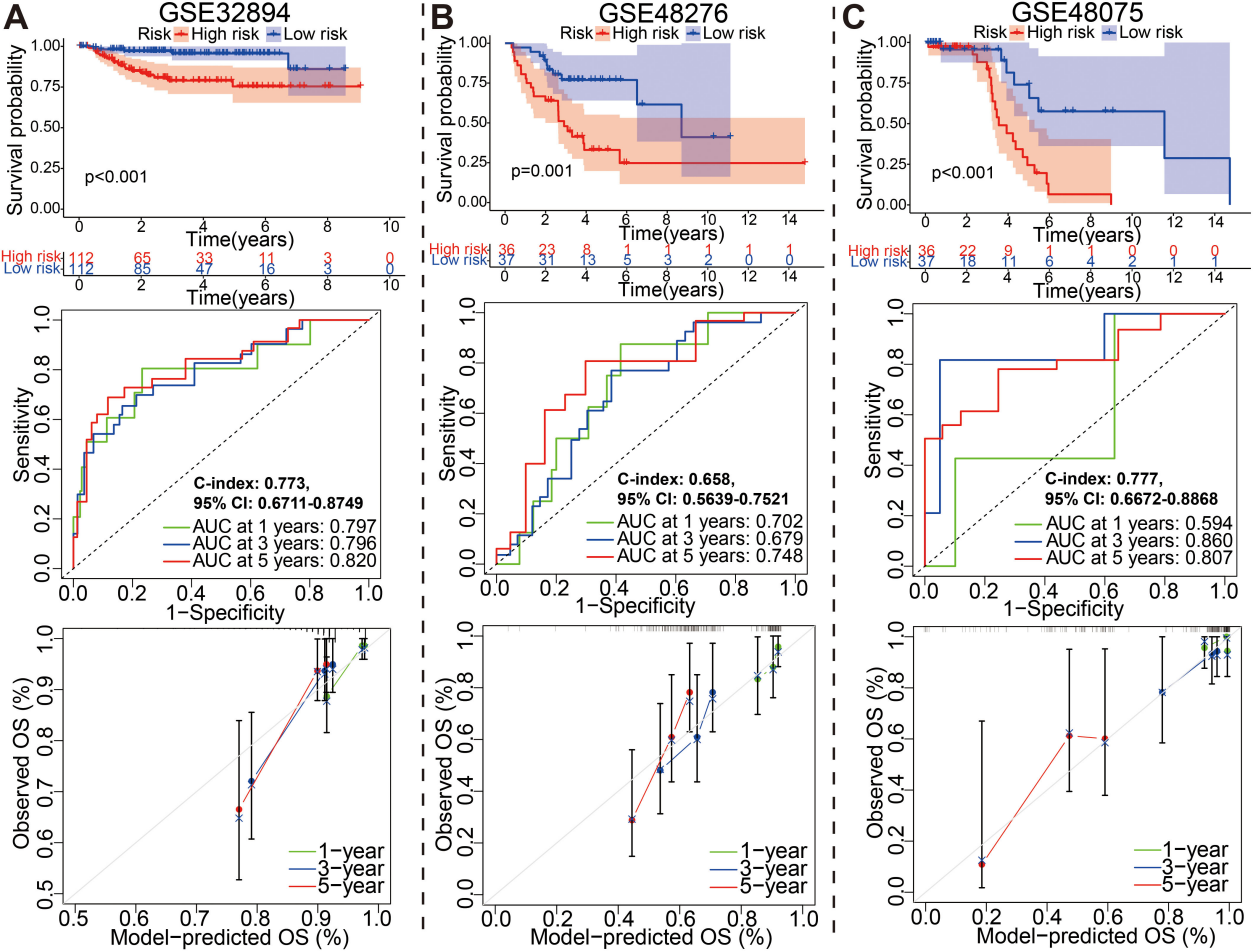
External identification of the prognostic model for bladder cancer based on **(A)** GSE32894, **(B)** GSE48276, and **(C)** GSE48075 datasets.

To evaluate the effectiveness and accuracy of the above 18 gene prognostic model in clinical practice, we conducted external validation using four independent datasets, including IMvigor210 GSE48075, GSE32894 and GSE48276 dataset from GEO database (**Figures 6**, **7**). It is worth mentioning that the IMvigor210 cohort, a cohort of 348 MIBC patients treated with Atezolizumab (PD-L1 inhibitor), was included to further evaluate the predictive capacity of the prediction models in BCa immunotherapy cohorts.

For IMvigor210 dateset, patients with high risk had a markedly worse OS than those with low risk based on the Kaplan-Meier curve (**Figure 6A**, P<0.001). Time-dependent ROC curves were used to assess the predictive performance of the risk score for OS, and the curve area (AUC) reached 0.679 at 10 months, 0.708 at 20 months (**Figure 6B**). The C-index based on the IMvigor210 set was 0.625 (95% CI, 0.5878–0.6622). Compared with the ideal model, the calibration plot for an OS of 10 months and 20 months was a good predictor (**Figure 6C**). The spread of risk scores were revealed for the bladder cancer samples from IMvigor210 cohort. Green indicates low risk, red indicates high risk. The spread of overall outcome was illustrated (**Figure 6D**). In green, the dots indicate a live patient, while in red, the dots indicate a dead patient. It is evident that bladder cancer patients with higher risk scores have a worse prognosis (**Figure 6E**). The heatmap showing expression of these 18 genes in the two groups (**Figure 6F**). And, the wilcox rank-sum test and chi-square test was also adopted to evaluate the correlations between the risk score group and available clinical variables (**Figures 6G, H**). Obviously, there were significantly associations between risk score/group and treatment response, metastatic state, immune phenotype, or TCGA subtype. It is worth saying that PD/SD patients exhibited higher risk scores than CR/PR patients, and patients with high-risk were almost all PD/SD cohort (89%).

Similarly, for GSE32894, GSE48276, and GSE48075 datasets, the KM, ROC and calibration curves analysis results in all three external validation sets were highly consistent with those of the training or above-mentioned validation sets; namely, patients with the high-risk group exhibited a worse prognosis (**Figure 7**). Moreover, These graphs, including risk score distribution, living status, gene expression heatmap, were plotted in above these verification cohorts (**Supplementary Figure S12**). It is notable that the C-index eventually reached 0.773 (95% CI, 0.6711–0.8749), 0.658 (95% CI, 0.5639–0.7521), and 0.777 (95% CI, 0.6672–0.8868) for the GSE32894, GSE48276, and GSE48075 datasets, respectively. The results of all these validation sets also suggested that the 18 gene signature is a good prognostic factor of BCa patients with or without ever receiving immunotherapy.

### The correlation between the prognostic model and aforementioned glycolysis-related clusters, TMB, mapping of mutations as well as the tumor immunity

**Figure 8:**
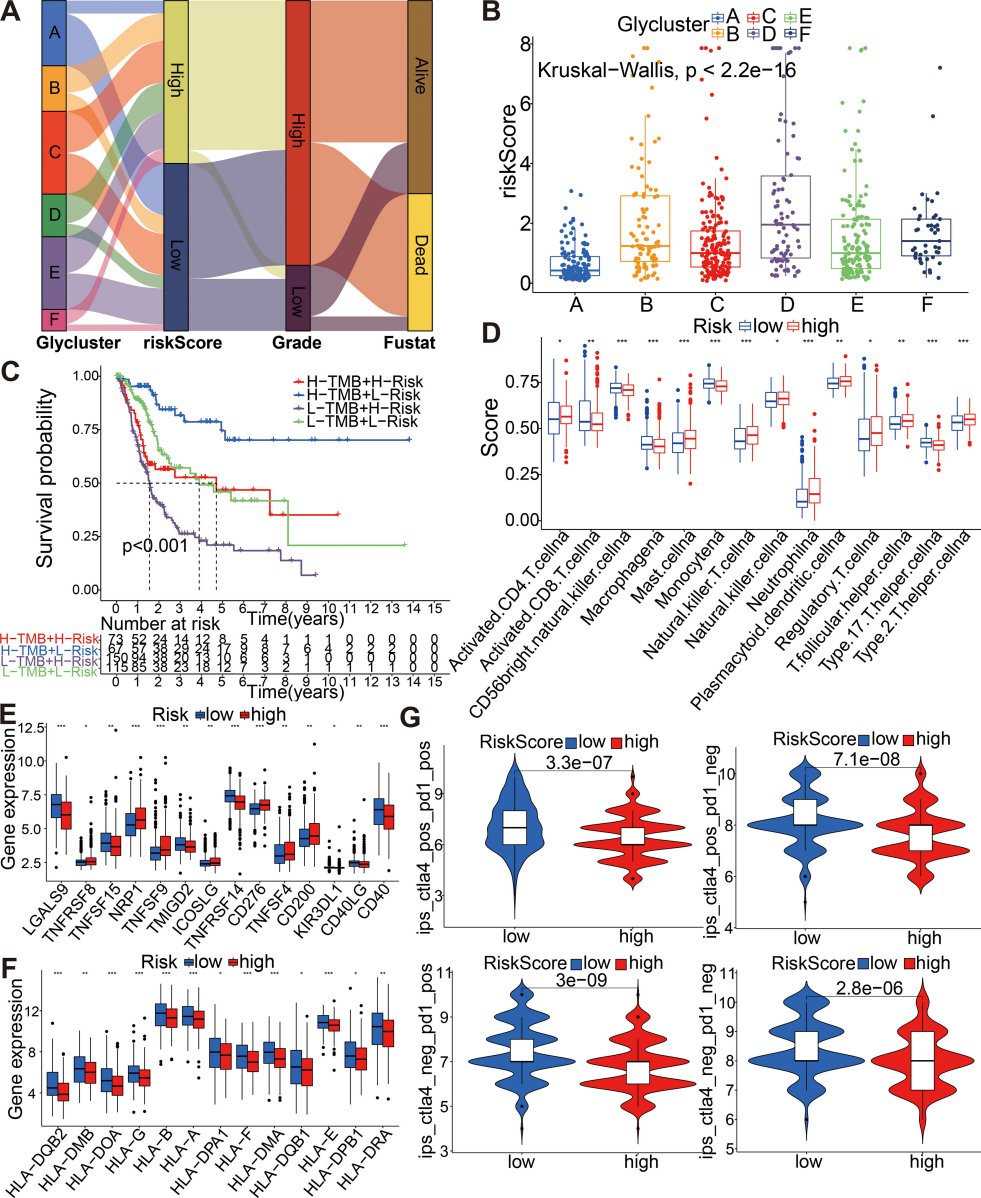
**(A)** Sankey diagram of glycolysis-related gene cluster distribution in groups with different risk score and survival outcomes. **(B)** The boxplot showing the relationship between the 6 clusters and risk score. **(C)** Kaplan–Meier curves for patients stratified by both TMB and risk score. **(D)** The difference in the expression of immune cell between high and low risk score. **(E, F)** The difference in the expression of immune checkpoint-related genes and HLA genes between high-risk and low-risk groups. **(G)** The modeled potential application values of ICBs for BCa. *p < 0.05, **p < 0.01, ***p < 0.001.

The Sankey diagram fully illustrated the association between glycolysis-related clusters, model risk score group, and clinical characteristics (**Figure 8A**). The boxplot was used to understand the relationship between the six above-mentioned glycolysis-related clusters and risk score (**Figure 8B**). As shown in the boxplot, the risks core was dramatically higher in the cluster D, while showed a dramatically lower level in the cluster A (Kruskal-Wallis test, p< 2.2e−16). Then, we further subdivided patients into 4 subgroups including high TMB plus high risk score, low TMB plus high risk score, high TMB plus low risk score and low TMB plus low risk score to assess the synergistic effects of TMB and risk score in bladder cancer. The high TMB plus low risk score subgroup displayed better survival than the high TMB plus high risk score subgroup (log-rank test, p < 0.001) (**Figure 8C**). To explore the relationship between risk group and immune status, we quantified immune-associated enrichment scores using ssGSEA algorithm. All the score of 14 immune cell, especially the score of CD56-bright-natural-killer-cellna, Macrophagena, Type-2-T-helper-cellna, Monocytena, Natural-killer-T-cellna, Neutrophilna, Type-17-T-helper-cellna and Mast-cellna were significantly different between the low and high risk group (all adjusted P<0.05, **Figure 8D**). Furthermore, we examined somatic variants of driver genes between low- and high-risk groups using the R package “maftools”. An analysis was carried out on the top 20 driver genes with the highest mutation frequency in high and low risk groups (**Supplementary Figure S13**).

### The potential role of modeled genes in predicting the immunotherapeutic efficacy in multiple independent immunotherapy cohorts

A comparison was made between high risk and low risk groups for the expression of immune checkpoint-related genes and HLA genes by wilcox ranksum test. We found significantly different expression of most immune checkpoint-related genes and HLA genes between the two groups, such as LGALS9, NRP1, TNFSF9, TNFRSF14, CD276, CD40, HLA-DQB2, HLA-DOA, HLA-G, HLA-B, HLA-A, HLA-F, HLA-DMA, and HLA-E (**Figures 8E, F**). These results suggested that the glycolysis-associated gene signature was associated with the regulation of tumor immune checkpoints. Afterward, we used the scores of IPS, IPS with CTLA4 blocker, IPS with PD1 blocker, and IPS with CTLA4 plus PD1 blocker to assess the modeled potential application values of ICBs for BCa; and IPS score in low-risk group was significantly higher compared with that in the high-risk group (**Figure 8G**).

**Figure 9:**
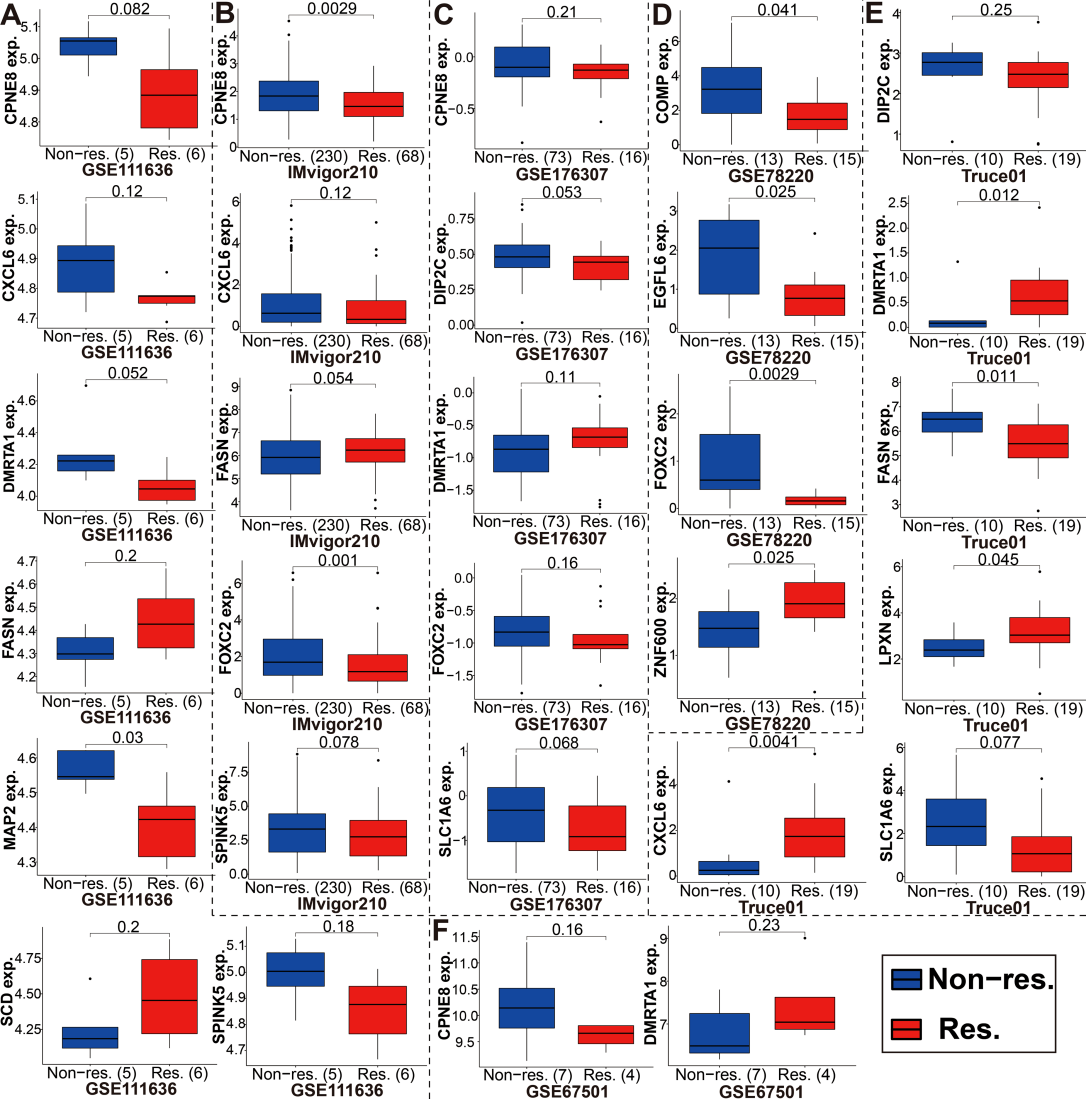
The expression level of the modeled genes in groups with a different immunotherapy response status. **(A-F)** These modeled genes expression in different cohorts, including GSE111636, Imvigor210, GSE176307, GSE78220, our mRNA sequencing (TRUCE-01), and GSE67501.

**Figure 10:**
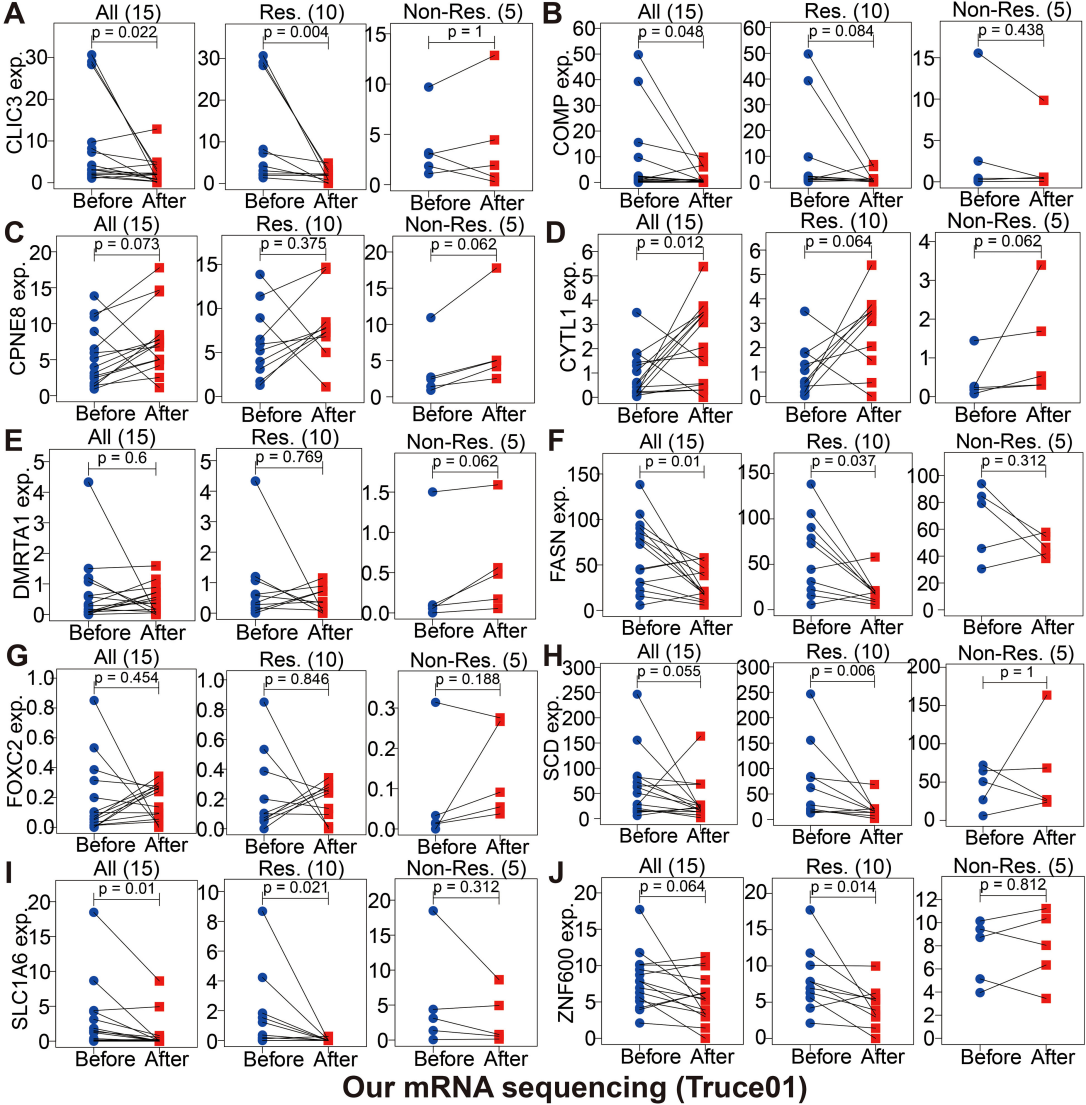
The changes of partially modeled genes expression before and after tislelizumab combined with low-dose nab-paclitaxel (TRUCE-01) of bladder cancer **(A-J)**.

Immunotherapy using anti-PD-1/PD-L1 have undoubtedly made a great breakthrough in cancer treatment. Thus, we conducted the association analyses between modeled genes expression and effectiveness of immunotherapy in authenticity population of bladder cancer (IMvigor210, GSE111636 and GSE176307), melanoma (GSE78220), renal cell carcinoma (GSE67501), and our mRNA sequencing (Truce01). Patients were divided into two groups as the low and high expression groups by the median expression level of modeled genes. We identified that the expression level of CPNE8/FOXC2/SPINK5/CXCL6 was negatively correlated with objective responses to anti-PD-L1 or anti-PD-1 treatment, and patients with responses to immunotherapy presented lower expression in the Imvigor210, GSE111636, GSE176307, GSE78220 or GSE67501 datasets (**Figure 9**). Conversely, we also found that the expression level of DMRTA1/SCD/LPXN was positively correlated with responses to immunotherapy in the GSE67501, GSE111636, GSE176307 or our mRNA sequencing cohorts (**Figure 9**). Moreover, in our ongoing single-arm phase II clinical study (TRUCE-01, NCT04730219), we also illustrated that the expression level of CLIC3/COMP/FASN/SCD/SLC1A6/ZNF600 in BCa cases with response to tislelizumab combined with nab-paclitaxel therapy significantly decreased after treatment, reversely the expression level of CPNE8/CYTL1/DMRTA1/FOXC2 increased after treatment in non-responsive cases (**Figure 10**). In all, these results show that expression of modeled genes can help predict immunotherapy response.

### Exploration of the tumor immune microenvironment and chemosensitivity based on the glycolysis-associated gene prognostic model

**Figure 11:**
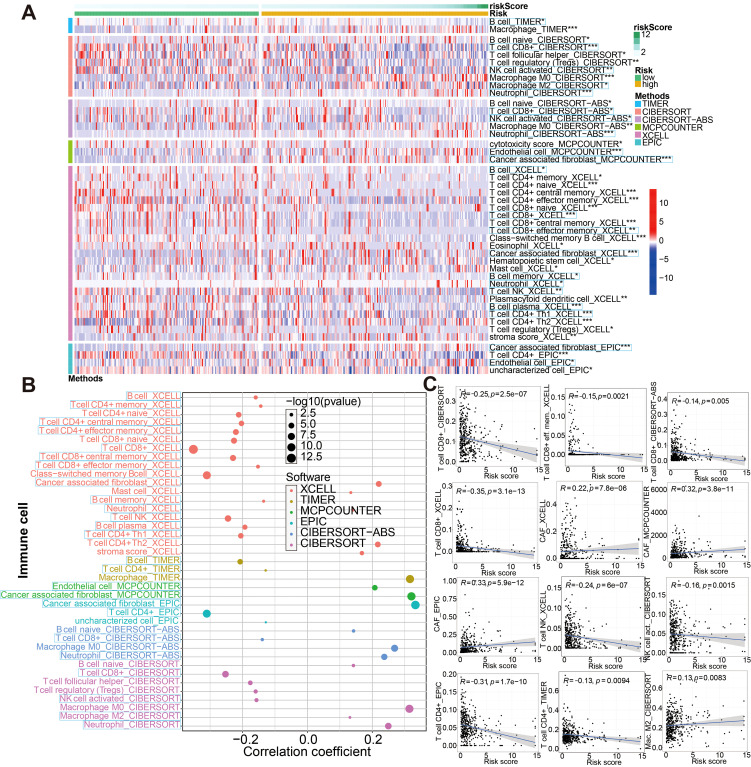
Associations between glycolysis-related gene signature and immune-cell infiltration evaluated by seven different approaches. **(A)** There were significant differences in some immune infiltrate components between low- and high-risk groups. **(B, C)** Correlation analysis between the riskscore and infiltrating immune cells abundance. *P < 0.05, **P < 0.01 and ***P < 0.001.

Tumor progression, prognosis, and therapeutic effect, especially with immunotherapy, are dependent on the level of immune cell infiltration in tumor microenvironment (TME). According to the 7 immune-infiltration algorithm, we studied the correlation between risk score and immune cell infiltration. A heatmap of 45 significant differences of immune cells including CD8+ T cells, cancer associated fibroblast, B cell, macrophage M2, neutrophils, NK cell and endothelial cell and so on in the two risk groups is presented in **Figure 11A** (Wilcoxon test, P < 0.01). Meanwhile, using spearman’s rank correlation analysis, we compared riskscore and immune-infiltration cells (**Figure 11B**). And in **Figure 11C**, the linear fit between riskscore and immune cells that affect immunotherapy is further depicted. Beside this, the ssGSEA immune-cell score from merge and IMvigor210 cohorts was implemented to investigate whether riskscore is associated with immune cell enrichment in BCa; our signature showed a significant negative correlation with CD8+ T cells infiltration (**Supplementary Figure S14**). That argument were consistent with the above conclusion. These results suggested that the glycolysis-associated gene signature was associated with the regulation of tumor immune checkpoints.

In order to further prove that our glycolysis-related risk score has good reliability, we studied the drug sensitivity of the compounds picked out from glycolysis-related 18 genes of this model. A transcriptome analysis of 60 cancer cell lines collected from the CellMiner database was used to screen out these compounds using our 18 model genes. Next, we examined the correlation between 18 model genes expression and IC50 for each drug type (**Supplementary File S8**), and thus screened out top 20 most significant IC50-associated agents (**Supplementary Figure S15A**) and several clinically commonly used drugs (including Cisplatin, Docetaxel, Doxorubicin, Paclitaxel, Gemcitabine, Methotrexate and Vinblastine) presented in **Supplementary Figure S15B**. Also, by using the pRRophetic algorithm, we determined the IC50s of four commonly used chemotherapeutic drugs that treat BCa. Low-risk group had a relatively high effect on these drug sensitivity tests compared with high-risk group (P<0.05, wilcox test; **Supplementary Figure S15C**). Further, we conducted a spearman analysis between the risk score and the IC50 of the chemotherapy agents listed above. As evident from **Supplementary Figure S15D**, correlation analysis results matched those of Wilcox analysis of the risk groups mentioned earlier (P<0.05, spearman correlation test).

### The model-associated partial genes expression validation by qRT-PCR and the HPA database

**Figure 12:**
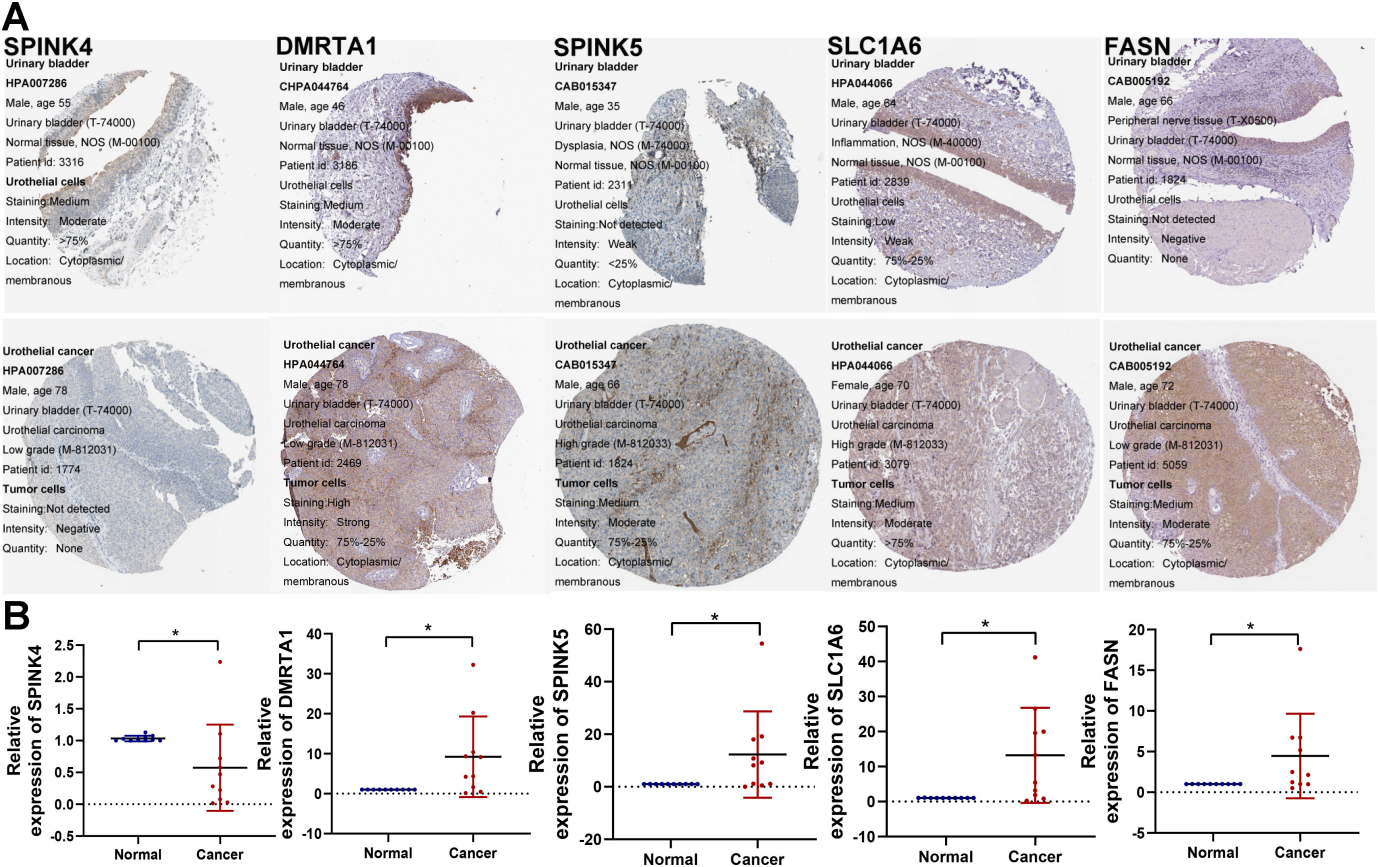
Validation of model-associated gene expression. **(A)** The protein expression levels of the glycolysis-related partial model genes were confirmed by the HPA database. **(B)** qRT-PCR was utilized to detect the expression of SPINK4, SPINK5, DMRTA1, SLC1A6, and FASN in 10 paired tumor tissues. *P < 0.05.

Subsequently, we analyzed the protein expression levels of several genes of interest using the Human Protein Atlas (HPA). Among them, SPINK5, DMRTA1, SLC1A6, and FASN were upregulated in BCa tissues. SPINK4 were downregulated compared to normal tissues, as shown in **Figure 12A**. Furthermore, the mRNA expression levels of these genes were detected in 10 pairs of BCa tissues and corresponding normal tissues. The qRT-PCR results showed the similar trend as the above protein results, as shown in **Figure 12B**.

### The biological behavior of FASN *in vitro* experiments

**Figure 13:**
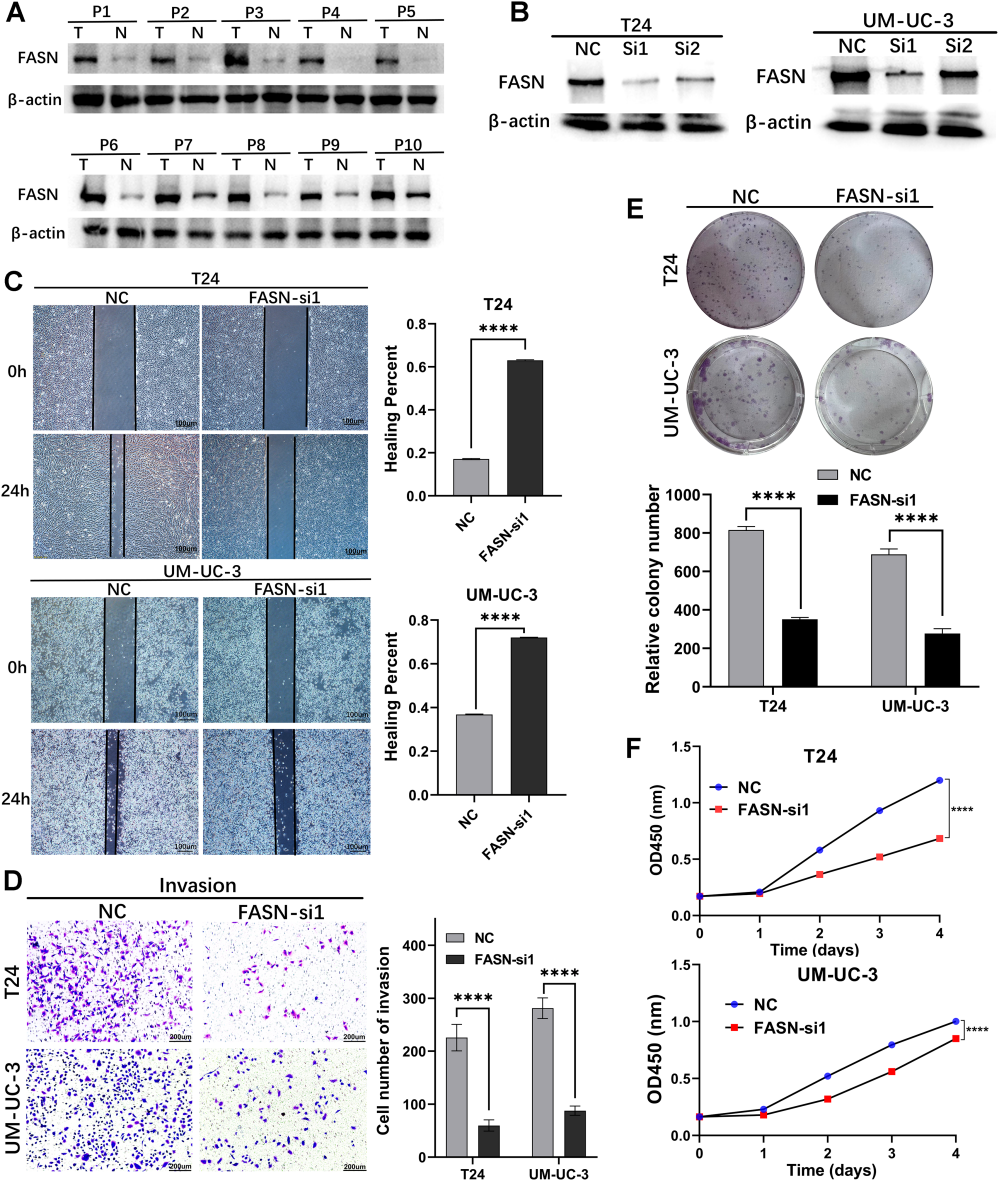
Loss‐of‐function experiments were conducted to explore the biological function of FASN *in vitro*. **(A)** WB assay to examine the FASN protein expression in BCa tissues (T) and normal bladder tissues (N), β-actin protein served as control. **(B)** FASN small-interfering RNA (siRNA) transfection efficiency was assessed by WB in T24 and UM-UC-3 cells. **(C)** The wound healing assay demonstrated the capacity of migration in T24 and UM-UC-3 cells. **(D)** The transwell assay demonstrated the capacity of invasion in T24 and UM-UC-3 cells. **(E)** The colony-forming assay detected the proliferation ability of tumor cells. **(F)** CCK-8 assays were utilized to detect cell proliferation ability in T24 and UM-UC-3 cells. ****p < 0.0001.

To investigate the biological function of FASN, we performed *in vitro* experiments using BCa cells. In the beginning, we assessed FASN expression in 10 bladder cancer tissue specimens using western blotting (WB), revealing higher expression levels in tumors compared to adjacent noncancerous tissues (**Figure 13A**). Subsequently, we explored the functional impact of FASN on the biological behavior of BCa cells. T24 and UM-UC-3 cells were transiently transfected with siRNAs targeting FASN or control siRNAs for 48 hours, and transfection efficiency was confirmed by WB (**Figure 13B**). Specifically, we evaluated the effect of FASN knockdown on the migration ability of T24 and UM-UC-3 cells using wound healing and transwell migration assays. Our results demonstrated that FASN knockdown significantly inhibited the migration and invasion ability of these cell lines (**Figures 13C, D**). Furthermore, we assessed the effect of FASN knockdown on T24 and UM-UC-3 cell proliferation using colony formation and CCK-8 assays, revealing a significant suppression in proliferation compared to controls (**Figures 13E, F**).Collectively, these findings indicate that FASN plays a crucial role in promoting proliferation, migration, and invasion of BCa cells.

## Discussion

In many cases, patients who are diagnosed with bladder cancer have advanced disease at the time of diagnosis, so they miss the ideal treatment period, and their prognosis is poor. Currently, the International Union Against Cancer (UICC) and International Society of Urological Pathology (ISUP) are the most common criterion used to determine bladder cancer prognoses. Patients may still experience different outcomes at the same stage because their tumors may have different molecular characteristics. It is therefore imperative that a more sensitive prognostic diagnostic method is developed that is based on the molecular characteristics of bladder cancer patients. This study aimed to identify molecular signatures that can aid in predicting prognoses and evaluating the efficacy of immunotherapy. For this study, we analyzed the mRNA expression and clinical data of BCa in a public database and identified 34 glycolysis-related DEGs associated with prognosis. Based on the 34 genes, we divided the samples into 6 clusters and found that cluster A had the best prognosis, while cluster D had the worst prognosis. Subsequently, 29 immune cells were also analyzed for their levels of infiltration by ssGSEA algorithm. We found that the infiltration of CD56dim-natural-killer-cellna, Monocytena, and Type17-T-helper-cellna were dramatically higher in cluster A than in cluster D. Some studies have pointed out that tumor progression and blood-borne metastasis could be inhibited by the CD56dim natural killer cells within the immune system (25). In addition, cytokines appear to direct monocytes to kill malignant cells by inducing cell death and phagocytosis (26). Some researchers point out Type17-T-helper-cellna in the tumor correlated with reduced tumor progression and improved patient survival (27). These views may be helpful to introduce the reason why cluster A had the best prognosis.

Afterward, we established a Glycolysis-related 18-gene signature in order to more accurately predict prognosis of BCa patients in the era of precision medicine based on the fused merge cohort (including TCGA, GSE13507 and GSE31684) through LASSO regression. Among these selected partial genes, the protein or mRNA expression of them was confirmed by the HPA database and qRT-PCR. The 18 genes were CPNE8, CXCL6, COMP, SPINK4, HLA-DQB2, CLIC3, DMRTA1, MAP2, ZNF600, CYTL1, DIP2C, FOXC2, LPXN, SPINK5, SLC1A6, FASN, SCD and EGFL6. This predictive signature has a high prediction consistency, discrimination capability, and accuracy. As a side note, the model has been validated in several external cohorts of BCa (IMvigor210, GSE32894, GSE48276 and GSE48075). Based on Kaplan-Meier analysis, low-risk patients had a promising survival advantage. And, according to the test in the merge training set, internal validation set, and the four external testing sets, we found that this model had excellent accuracy. Furthermore, we explored the relationship between gender, age, grade, T stage, N stage and the modeled risk score/groups. There was a significant increase in riskscores among BCa patients with high-grade, T3/4, N1-3, immune-excluded, liver metastases or the immunotherapy ineffective-response (SD/PD) as compared with oppositional traits. So that, these features were incorporated into a nomogram which could be used to predict clinical survival.

Therefore, we performed a systematic investigation analysis of these genes among the model. A member of the Copine family, CPNE8 contributes to the development of a variety of tumors. Ovarian clear cell carcinoma is dramatically inhibited by CPNE8 knockout studies (28). In other study, prognosis was related to high expression of CPNE8 in gastric cancer (29). Cancers such as prostate cancer, gastrointestinal tumors, and breast cancer appear to be mediated by CXCL6 (30). A cartilage metabolism marker, COMP modulates the cellular phenotype during tissue genesis and remodeling. COMP has been shown to promote the progression of breast, colon, and prostate cancers in recent studies (31–33). SPINK was originally composed of four members in humans and belongs to the Kazal type of serine protease inhibitor family (34). A recent study showed that serum SPINK4 levels were elevated in CRC preoperatively, decreased after resection, and were associated with distant metastases (35). The HLA-DQB2 gene has a limited polymorphism, an unknown function, and at least two transcription variants: in version 1, the full-length beta-chain is encoded, while in version 2, exon 4 is absent, which allows soluble proteins to be generated. There is immunological significance to HLA-DQB2 genes that have poor polymorphism, according to some observations (36). CLIC3, an intracellular chloride channel with 236 amino acids, resides at 9q34.3. Chloride ions are promoted by encoded proteins, which have a size of 26.6 ku (37). The prognosis is poorer for BCa patients with high levels of CLIC3 mRNA, as well as adverse clinicopathological characteristics. Using CLIC3, prognostic biomarkers of BCa can be identified (38). Genes encoded by the DMRT family play an important role in sexual development. There is a correlation between bladder cancer prognosis and DMRTA1 expression (39). MAPs are proteins associated with microtubules, which consist mainly of tubulin (40). Neurons express MAP2 and its subcellular distribution is particularly pronounced in dendrites and soma (41). In both vitro and *in vivo*, studies have demonstrated that MAP2 expression can reduce melanoma cell growth and proliferation (42). An important transcriptional regulator, ZNF600, is a zinc finger protein. New loci of phospholipids have been associated with ZNF600 in recent GWAS studies (43). A novel cytokine called CYTL1 has been detected in CD34+ hematopoietic cells for the first time. As CYTL1 is hypermethylated in breast, lung and stomach cancers, its expression is significantly reduced in these cancers. As a result of decreasing STAT3 phosphorylation, CYTL1 could inhibit tumor metastasis (44). From worm to human, DIP2C is a member of the disconnected interacting protein 2 (DIP2) family and plays an important role in fatty-acid metabolism (45) and methylation machinery (46). In lung cancer tissues (47) and breast cancer cells (48), DIP2C was found to be significantly mutated. As a result of its somatic mutations, the protein function in breast cancer tissues might be affected, which is thought to be involved in tumor development (49). Known as winged helices because of the butterfly-like appearance of their loops, FOX proteins are a family of transcription factors (50). The transcription factor FOXC2 plays a crucial role in both angiogenesis and lymphangiogenesis (51), and may contribute to increased pathological angiogenesis and neovascularization, which play a crucial role in the growth and progression of tumors (52). Paxillin protein family member LPXN possesses LIM domains and LD motifs as protein-protein interaction domains (53). Prostate cancer cells expressed LPXN, which regulated invasion and adhesion (54). Through the PI3K/AKT pathway, LPXN also stimulate the proliferation, metastasis, and angiogenesis of bladder cancer (55). SPINKs are the largest group of inhibitors of serine proteases (56). Spink5 encodes a serine protease inhibitor called LEKTI, which is associated with lymphoid epithelial cells (57). The Wnt/β-catenin signaling pathway can be inhibited by SPINK5 in order to act against esophageal cancer cells proliferation, migration, and invasion (58). In mammals, SLC1A6 is one of a family of transporters known as the SLC1A family, which includes the excitatory amino acid transporter EAAT1-EAAT5 and the alanine serine cysteine transporter ASCT1-ASCT2 (59). Cancer of the bladder urothelium can be predicted by the expression of SLC1A6 (60). In most normal tissues except for liver and adipose tissue, FASN turns acetyl-CoA and malonyl-CoA into FAs. Prostate cancer patients with FASN protein overexpression have poor biochemical survival (61). Through SCD, monounsaturated fatty acids (MUFAs) are generated that contribute to cell growth, survival, differentiation, metabolic regulation, and signal transduction. Cancers such as lungs, breasts, esophagus, bladders, and liver are overexpressed in SCD (62). A member of the epidermal growth factor superfamily (EGF), EGFL6 is involved in cell cycle regulation, proliferation, and differentiation. Tumor-associated endothelial cells express high levels of EGFL6, which controls the development of blood vessels during physiological and pathological angiogenesis (63).

Patients with bladder cancer were divided into high and low risk groups based on the above risk model. High risk patients had a worse prognosis. As a result of GO and KEGG analyses, we identified that high risk individuals were activated in pathways that involved ECM-receptor interaction. ECM-receptor interaction pathways play a key role in the removal of tumors, adhesion, degradation, movement, and hyperplasia. The ECM plays a role in tumor invasion and metastasis in multiple cancer types (64, 65). During colorectal cancer, the ECM may promote epithelial-mesenchymal transition (EMT) (66). Therefore, patients with high risk score may benefit from drugs that inhibit the migration and invasion of cancer cells by altering key adhesive protein expression patterns. Antigen processing and presentation pathways were activated in patients with low risk score. Therefore, the low risk group may benefit more from immunotherapeutic drugs. Apart from that, we also analyzed the sensitivity to chemotherapy agents commonly used for treating BCa by calculating IC50 value, and thereby picked out candidate small-molecule compounds.

Moreover, we also performed to assess the potential role of modeled genes as a biomarker for immunotherapy efficacy based on different immunotherapy cohorts (i.e., IMvigor210, GSE111636, GSE176307, GSE78220, GSE67501, and TRUCE-01). According to these immunotherapy datasets, we determined that the expression level of partial genes in the model, including CPNE8/FOXC2/SPINK5/CXCL6/DMRTA1/SCD/LPXN, etc., was remarkably associated with the efficacy of anti-PD-L1/anti-PD-1 treatment. Hargadon et al. reported that FOXC2 promotes melanoma progression via several oncogenic pathways, including xenobiotic metabolism, oxidative stress response and interferon responsiveness, as well as is a prognostic indicator of patient response to chemotherapy and immunotherapy (67). The immune-associated 7-IRG signature (containing gene SPINK5) constructed by Peng et al. could validly indicate survival prognosis and immunotherapy response of HCC patients (68). Previous research revealed that CXCL6 can enhance the growth and metastases of ESCC cells through activating STAT3/PD-L1 pathway (69); on the other hand, the CXCL6 secretion by breast cancer cells induced by Ionizing radiation that can recruit antitumor effector T cells, convert tumors into relatively “inflamed” peripheral tissues, and improve the effect of immunotherapy (70). Afterwards, based on our phase II clinical studies ongoing (term_id, TRUCE-01; registration number, NCT04730219), we identified that the expression level of CLIC3/COMP/FASN/SCD/SLC1A6/ZNF600 in BCa groups with CR/PR response to tislelizumab combined with low-dose nab-paclitaxel therapy significantly decreased after treatment, whereas the expression level of CPNE8/CYTL1/DMRTA1/FOXC2 were upregulated after treatment in SD/PD non-responsive cases. For ovarian cancer, aberrant activation of FASN oncogenic pathway cause the compromised antitumor immune response by lipid accumulation in tumor-infiltrating dendritic cells and then T-cells exclusion and dysfunction (71); and its inhibitors TVB3664 could be combined with other drugs for enhancing treatment efficacy of HCC (72). Overall, the expression patterns of 18 model-related genes displayed predictive accuracy and superb stability in detecting immunity-related characteristics or identifying immunotherapy response.

Apart from the above, since the model was initially associated with immune-related genes or the efficacy of immunotherapy, we then explored how it might be applicable to immune microenvironments. The results of 7 immune-infiltration algorithm analyses, indicated that the riskscore showed a significant positive correlation with the infiltration of cancer-associated fibroblasts (CAFs), macrophage M2, neutrophils, and endothelial cells, etc; nevertheless, riskscore was sharply and negatively associated with the infiltration level of CD8+ T, activated NK, and B cells. CAFs and macrophage M2 are the predominant cells within the tumor stroma and are responsible for maintaining a favorable microenvironment for tumor cell growth and proliferation (73–75); its can both block antitumor drugs as well as induce tumor resistance, which is closely related to poor prognoses (76, 77). On the other hand, cells such as CD8+ cytotoxic T cells and NK cells are capable of killing tumor cells (78, 79). In addition, high risk individuals infiltrated more immune cells according to ssGSEA algorithm analysis. The low risk group had higher levels of CD8+T cells, CD56bright natural killer cells, Macrophagena cells, Monocytena cells and Type17 T helper cells, and higher levels of immune checkpoint genes and HLA genes, indicating that immunotherapy may be beneficial for them.

In the present study, molecular typing of glycolysis genes is closely related to the prognosis of BCa patients. Subsequently, we established and validated a glycolysis-related gene signature as a predictive prognosis, immune infiltration, or drug sensitivity tool in BCa through integrated analysis of multiple data sets. Moreover, differential expression of these candidate model genes were next validated by real-time qRT-PCR and HPA database. The results also found that these genes of model may act as a promising biomarker for predicting the efficacy of immunotherapy in BCa patients. Finally, we also identified FASN as significantly contributing to BCa cells proliferation, migration and invasion via *in vitro* phenotypic experiments. Of course, to determine whether the risk model can accurately predict bladder cancer prognosis and immune response, more clinical samples and prospective studies are needed.

## Data Availability

The datasets presented in this study can be found in online repositories. The names of the repository/repositories and accession number(s) can be found in the article/**Supplementary Material**.

## Supplementary material

Supplementary Figure 1 Identification of the candidate glycolysis-related genes based on the TCGA cohort. **(A)** Venn diagram to identify 39 prognostic glycolysis-related DEGs between tumor and adjacent normal tissue. **(B)** Heatmap of expression profiles of 39 prognosis-associated DEGs. **(C)** Prognostic forest plots showing the results of the univariate Cox regression analysis of these 39 glycolysis-associated genes. **(D)** The PPI network analysis of these overlapping DEGs was performed based on the STRING database.

Supplementary Figure 2 The CNVs and mutations status for these filtered 34 glycolysis-related prognostic DEGs. **(A)** Frequency of copy number variations for each gene. **(B)** Location of the variant at chromosome per gene. Red squares indicates high level amplifications, blue denotes high level losses. **(C)** The waterfall plot shows the mutation distribution of these genes.

Supplementary Figure 3 GSVA enrichment analysis for KEGG pathway gene sets based on merge dataset. **(A-O)** The top 20 significantly enriched differences in pathway activities scored via GSVA of KEGG gene sets between every two subtyping group. (P) The intersection condition of differentially significant enriched pathways between every two clusters.

Supplementary Figure 4 Comparison of the ssGSEA enrichment scores for immune-related cells among different subtyping.

Supplementary Figure 5 UpSet plot showed the intersection status of differentially expressed genes between glycolysis-associated six clusters. The bar plot indicates the intersection size (number of genes).

Supplementary Figure 6 For differentially expressed genes among the six clusters, GO function and KEGG pathway enrichment analyses were conducted.

Supplementary Figure 7 Lasso regression further screen these prognosis-associated DEGs among the six clusters. **(A)** Lasso coefficient plot of candidate genes. **(B)** The tuning parameter (lambda) was chosen by cross validation.

Supplementary Figure 8 The relationship between the expression of the 18 model genes and the glycolysis-related six clusters established by us.

Supplementary Figure 9 Association analysis between risk score/group and clinicopathological data of BCa patients through wilcox rank test or chi-square test based on the merge dateset.

Supplementary Figure 10 Correlation analysis between the modeled risk score and accepted immunotyping results from the TCGA-BCa cohort.

Supplementary Figure 11 Stratification analysis of various clinicopathological factors by Kaplan–Meier curves for the patients with bladder cancer in the high and low risk score groups. Kaplan–Meier curves of OS in different subgroups stratified by **(A)** age, **(B)** stageT, **(C)** stage N, **(D)** gender, **(E)** grade, and **(F)** survival status.

Supplementary Figure 12 The risk score ranking, survival status scatter diagram, and expression heatmap of modeled genes in the **(A)** GSE32894, **(B)** GSE48276, and **(C)** GSE48075 cohorts.

Supplementary Figure 13 The oncoPrint in the low **(A)** and high **(B)** risk score groups based on TCGA-BCa cohort.

Supplementary Figure 14 Bubble diagram showed the spearman correlation between riskscore and the ssGSEA immune-associated enrichment scores from merge **(A)** and IMvigor210 **(B)** cohorts.

Supplementary Figure 15 Correlations between risk score/group and IC50 for different drugs. **(A)** The top 20 agents most significantly associated with model gene expression and **(B)** several clinically commonly used drugs by Spearman rank correlation test with p<0.05 using the CellMiner database. **(C)** Wilcox group analysis and **(D)** spearman correlation analysis all indicated that the glycolysis-related gene signature is robust to drug sensitivity of Cisplatin, Gemcitabine, Mitomycin C, and Doxorubicin from the pRRophetic algorithm.

Supplementary File S1 Clinical, histopathological and survival information of TCGA_BCa, IMvigor210 and five GEO datasets (i.e., GSE13507, GSE31684, GSE48276, GSE48075, and GSE32894).

Supplementary File S2 The efficacy information of immunotherapy from six immunotherapy cohorts, including IMvigor210, GSE111636, GSE176307, GSE78220, GSE67501, and Truce01.

Supplementary File S3 The pair-wise comparisons of glycolysis-related molecular typing in terms of GSVA score of KEGG pathways were performed based on merge datasets and these DE-pathways are summarised in this file.

Supplementary File S4 The pair-wise comparisons of glycolysis-related molecular typing in term of gene expression were performed based on merge datasets and these DE-genes are summarised in this file.

Supplementary File S5 GO function and KEGG pathway enrichment analyses were conducted for the 3754 DE-genes from the pairwise comparison between among the glycolysis-related subtyping in BCa.

Supplementary File S6 According to merge cohort, uniCox regression revealed 1223 molecular_typing-related DE-genes with significant prognostic significance.

Supplementary File S7 The GSEA enrichment was performed for the HALLMARK, KEGG and GO terms in high-risk *vs*. low-risk group based on merge datasets.

Supplementary File S8 Correlations between 18 model genes expression and IC50 for different drugs.
