# Supplementary figures and images for: Development and validation of a glycolysis-associated gene signature for predicting the prognosis, immune landscape, and drug sensitivity in bladder cancer
Source: Front Immunol. 2025 Jan 10;15:1430583. doi: 10.3389/fimmu.2024.1430583 (PMC11757262; doi:10.3389/fimmu.2024.1430583)

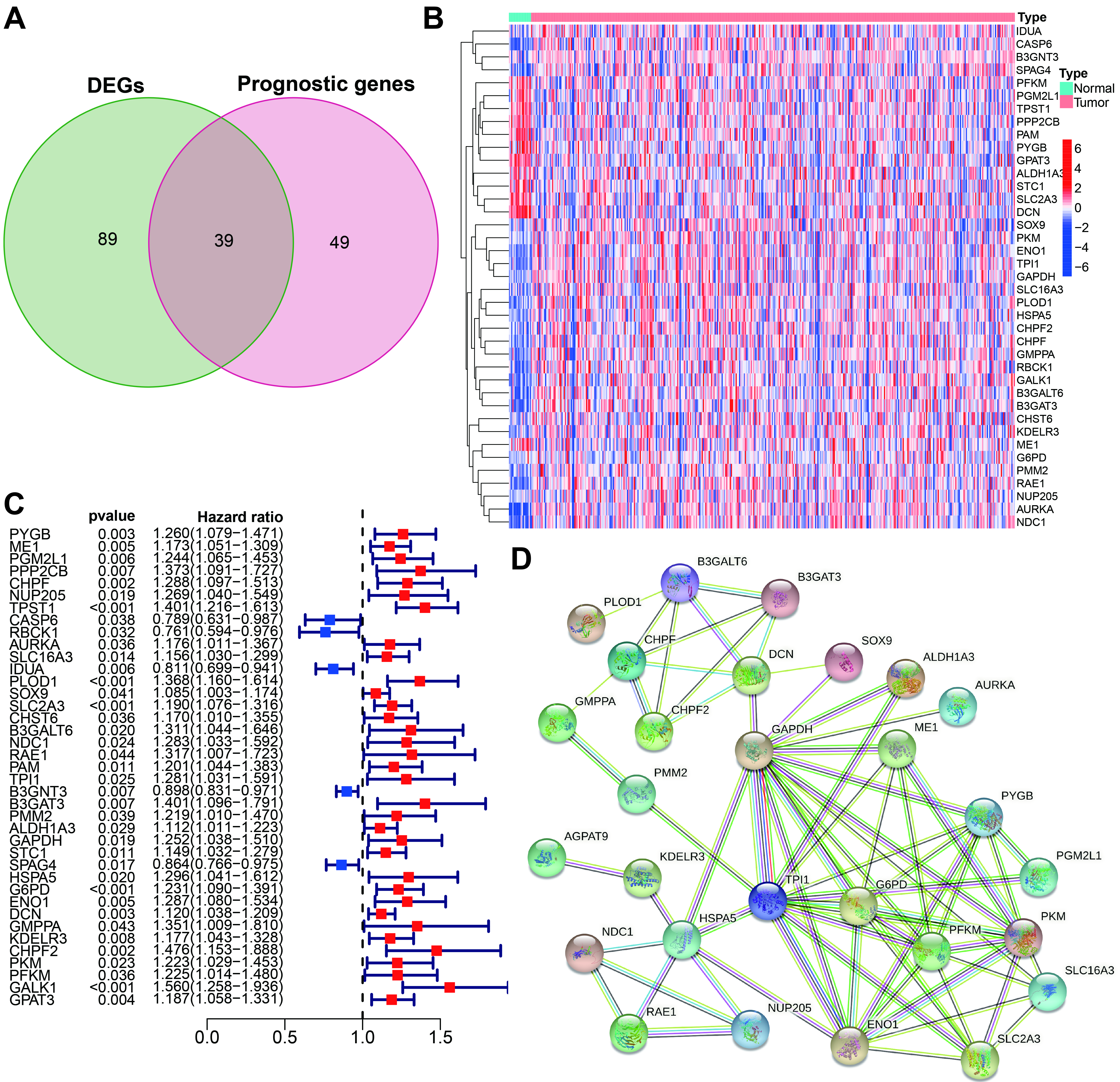

Supplement: Supplementary Figure 1 — Identification of the candidate glycolysis-related genes based on the TCGA cohort. (A) Venn diagram to identify 39 prognostic glycolysis-related DEGs between tumor and adjacent normal tissue. (B) Heatmap of expression profiles of 39 prognosis-associated DEGs. (C) Prognostic forest plots showing the results of the univariate Cox regression analysis of these 39 glycolysis-associated genes. (D) The PPI network analysis of these overlapping DEGs was performed based on the STRING database. [file Image1.tif]

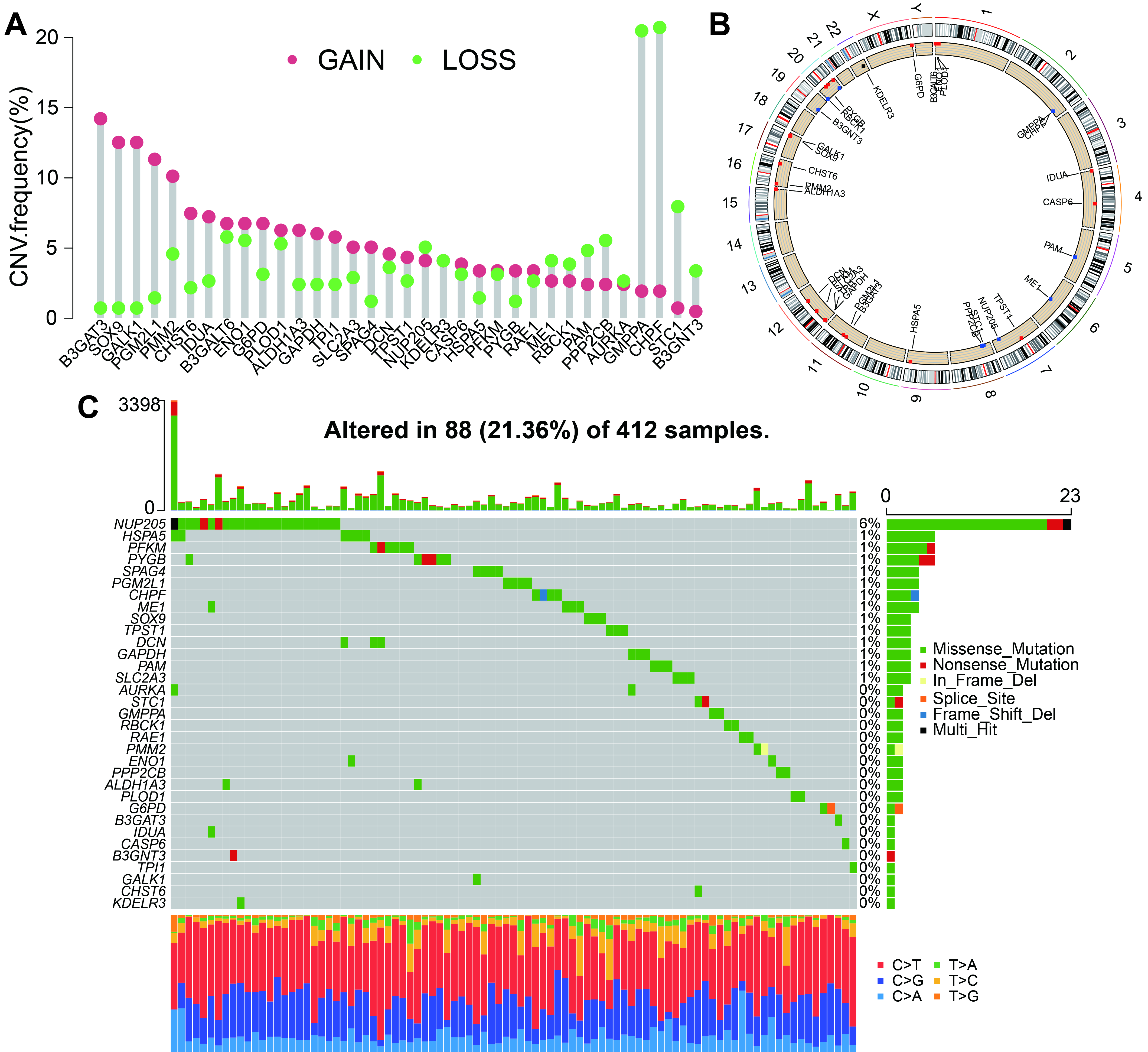

Supplement: Supplementary Figure 2 — The CNVs and mutations status for these filtered 34 glycolysis-related prognostic DEGs. (A) Frequency of copy number variations for each gene. (B) Location of the variant at chromosome per gene. Red squares indicates high level amplifications, blue denotes high level losses. (C) The waterfall plot shows the mutation distribution of these genes. [file Image2.tif]

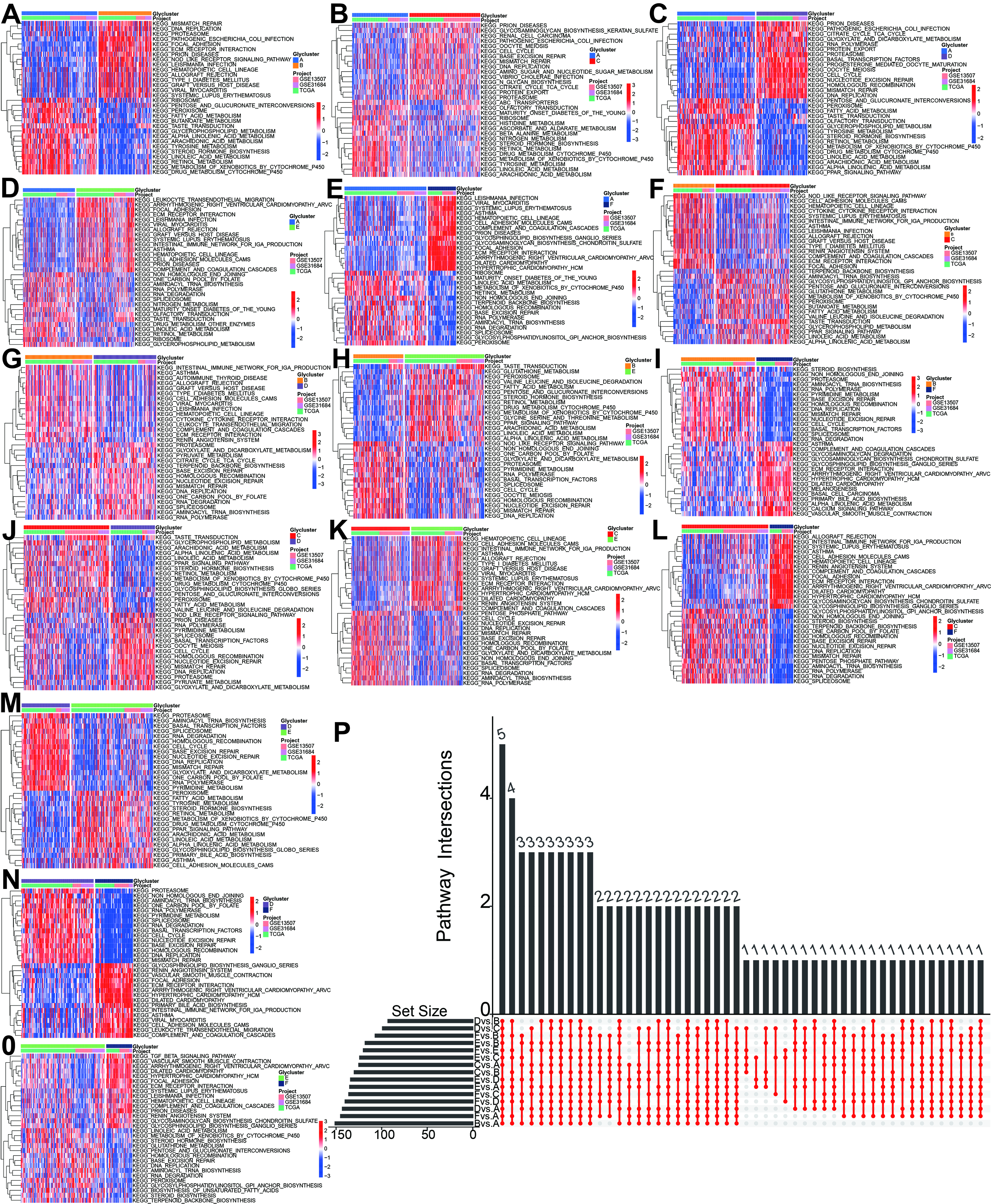

Supplement: Supplementary Figure 3 — GSVA enrichment analysis for KEGG pathway gene sets based on merge dataset. (A-O) The top 20 significantly enriched differences in pathway activities scored via GSVA of KEGG gene sets between every two subtyping group. (P) The intersection condition of differentially significant enriched pathways between every two clusters. [file Image3.tif]

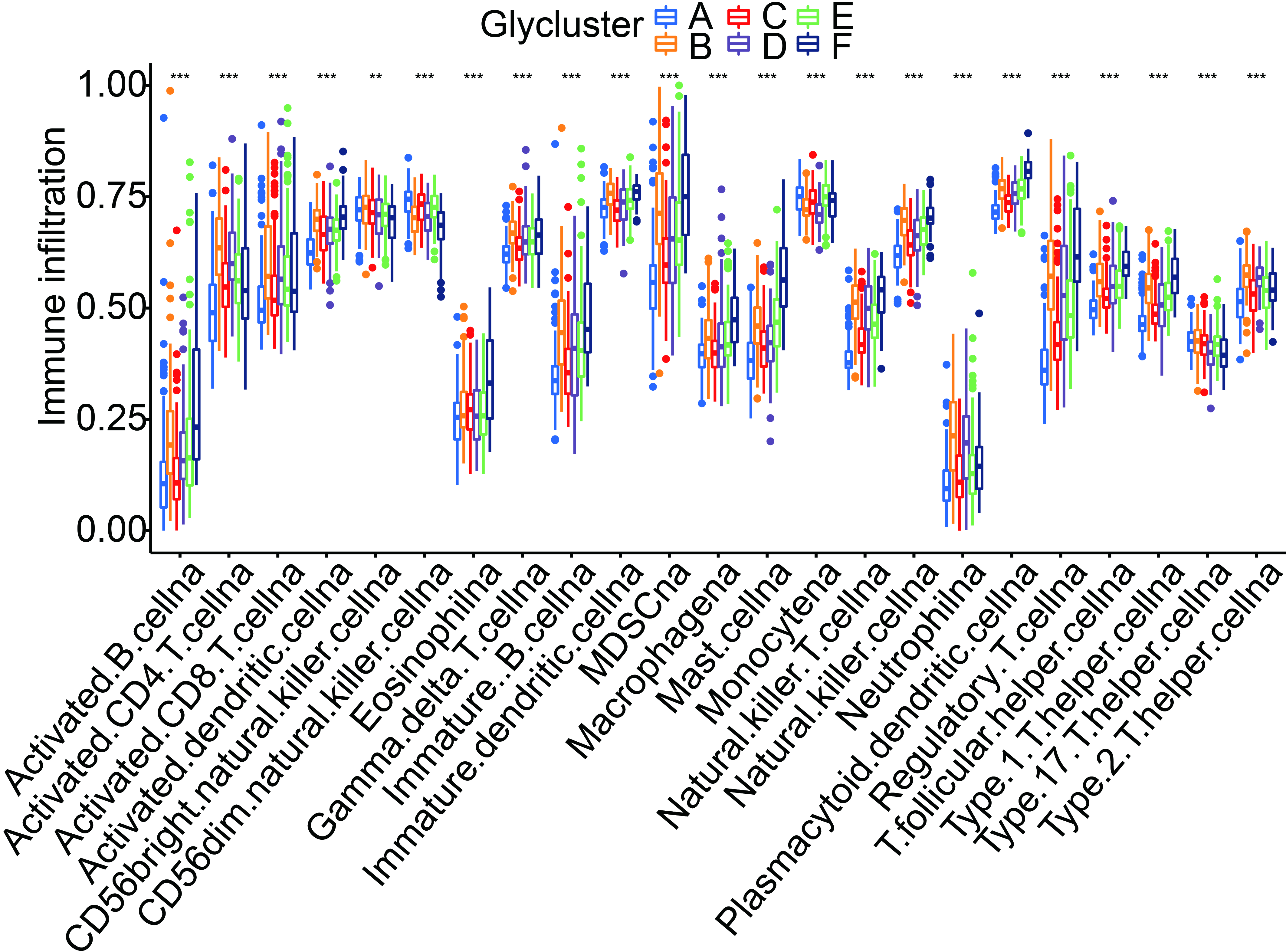

Supplement: Supplementary Figure 4 — Comparison of the ssGSEA enrichment scores for immune-related cells among different subtyping. [file Image4.tif]

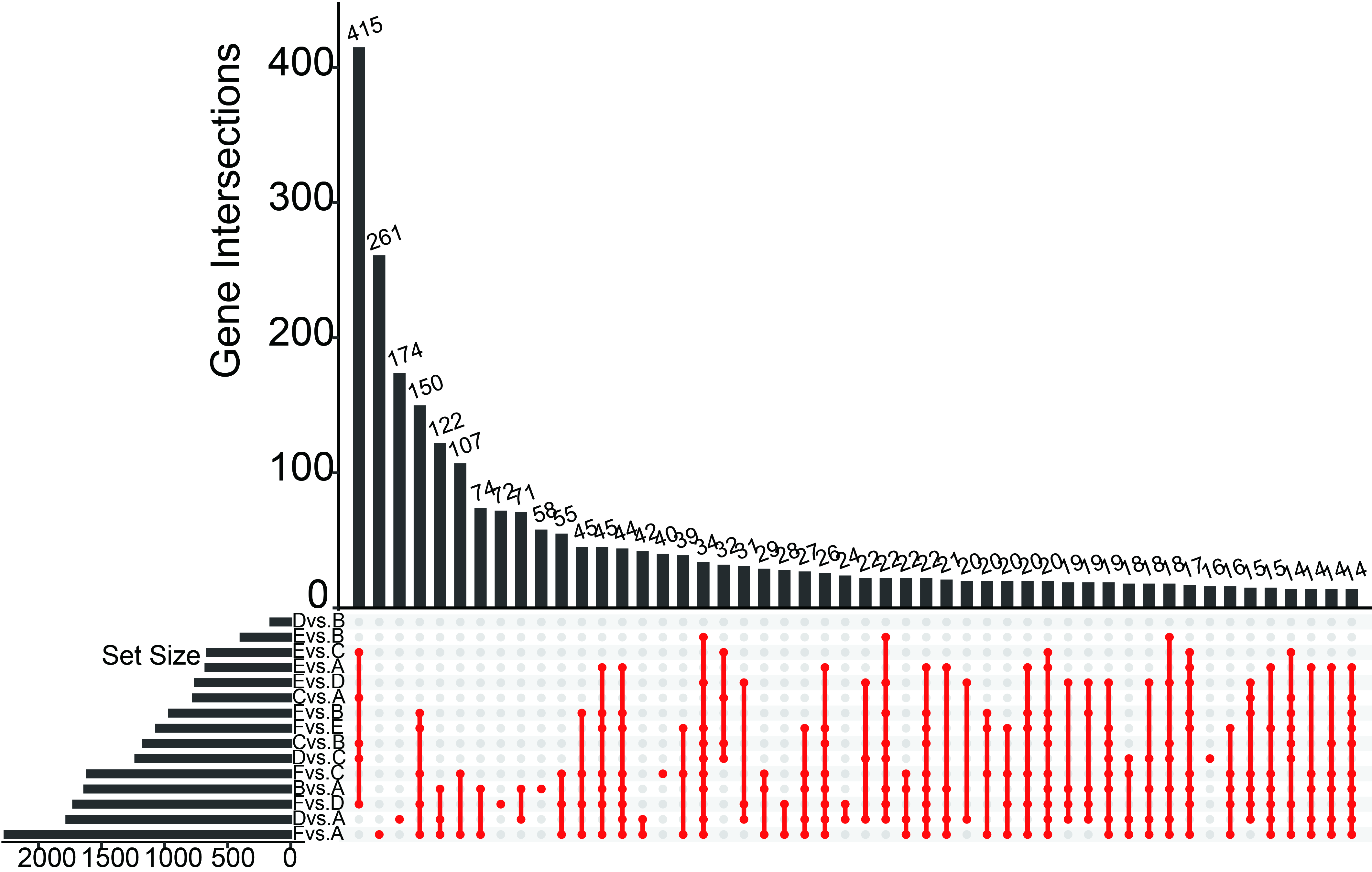

Supplement: Supplementary Figure 5 — UpSet plot showed the intersection status of differentially expressed genes between glycolysis-associated six clusters. The bar plot indicates the intersection size (number of genes). [file Image5.tif]

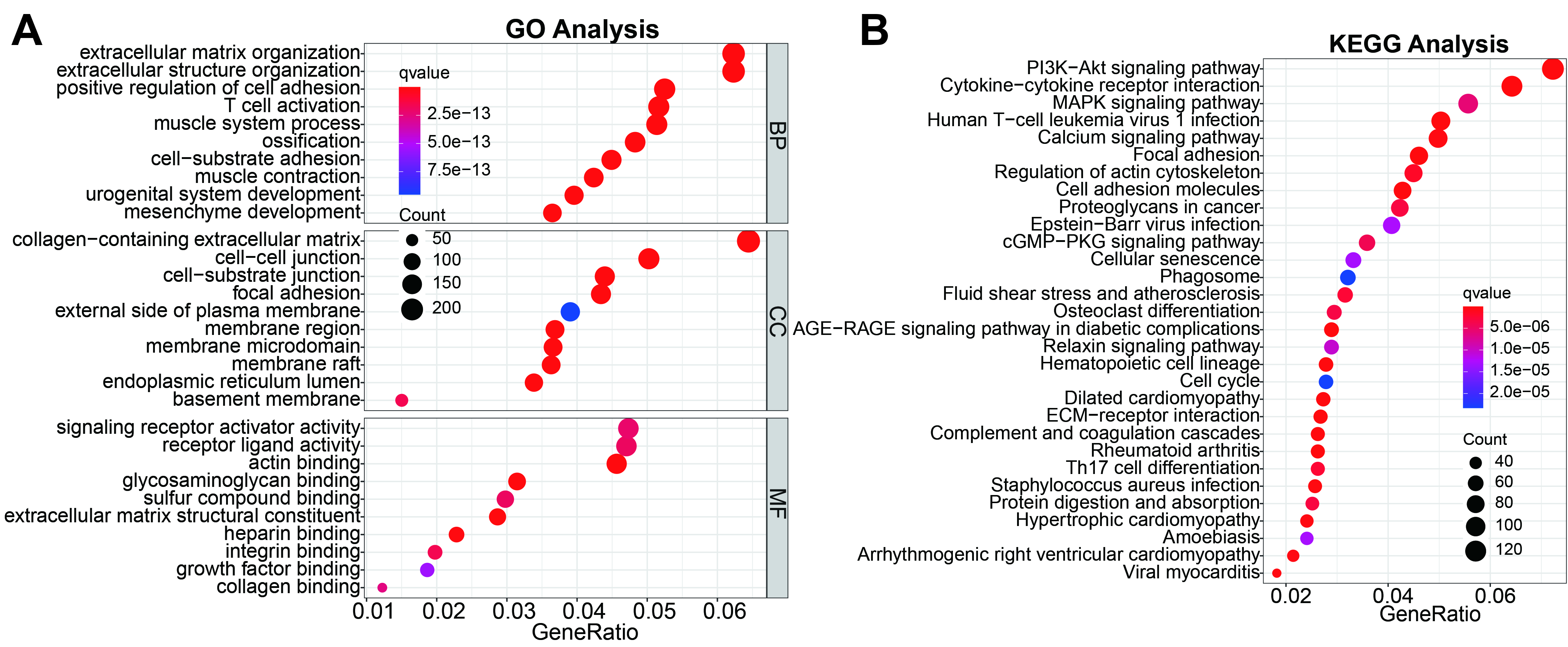

Supplement: Supplementary Figure 6 — For differentially expressed genes among the six clusters, GO function and KEGG pathway enrichment analyses were conducted. [file Image6.tif]

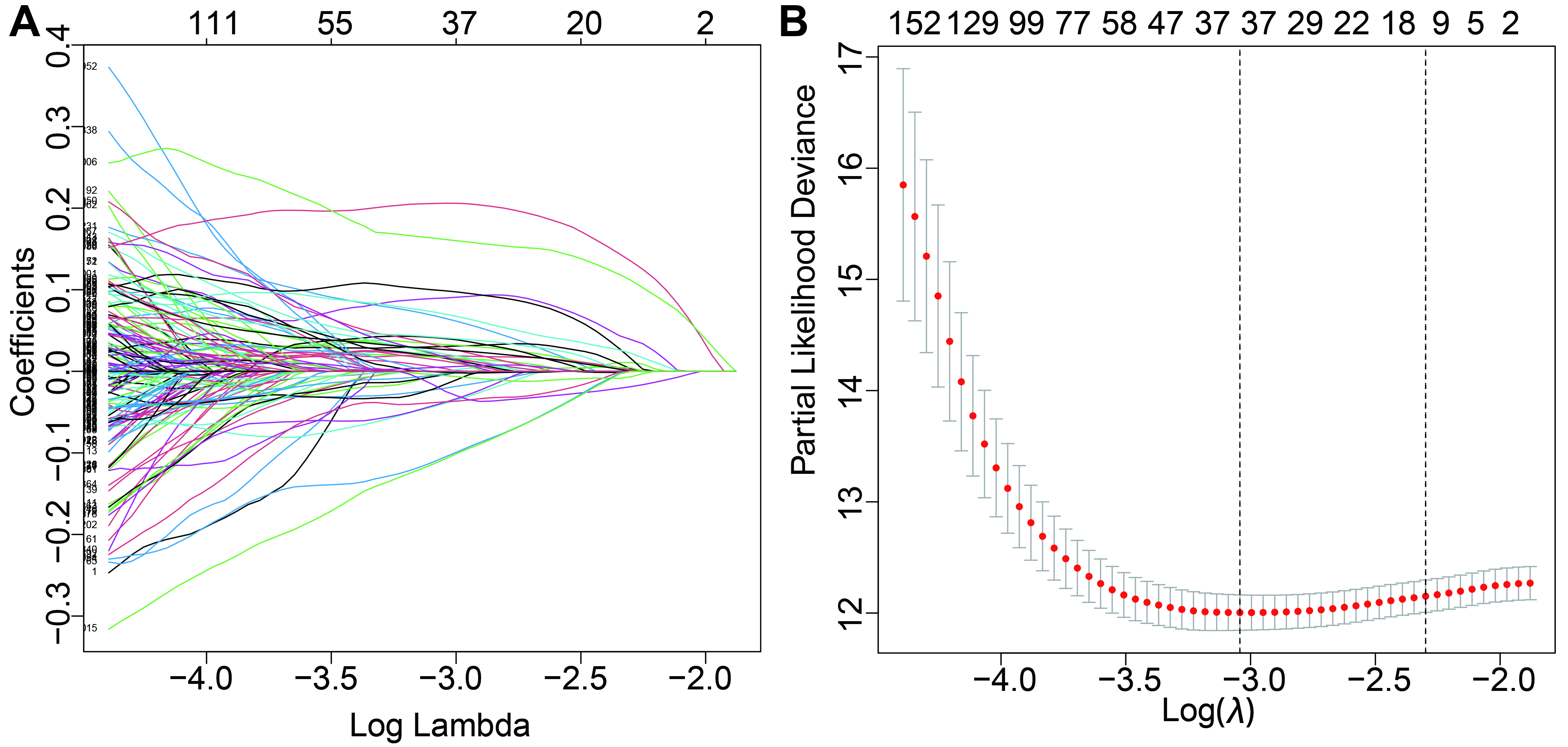

Supplement: Supplementary Figure 7 — Lasso regression further screen these prognosis-associated DEGs among the six clusters. (A) Lasso coefficient plot of candidate genes. (B) The tuning parameter (lambda) was chosen by cross validation. [file Image7.tif]

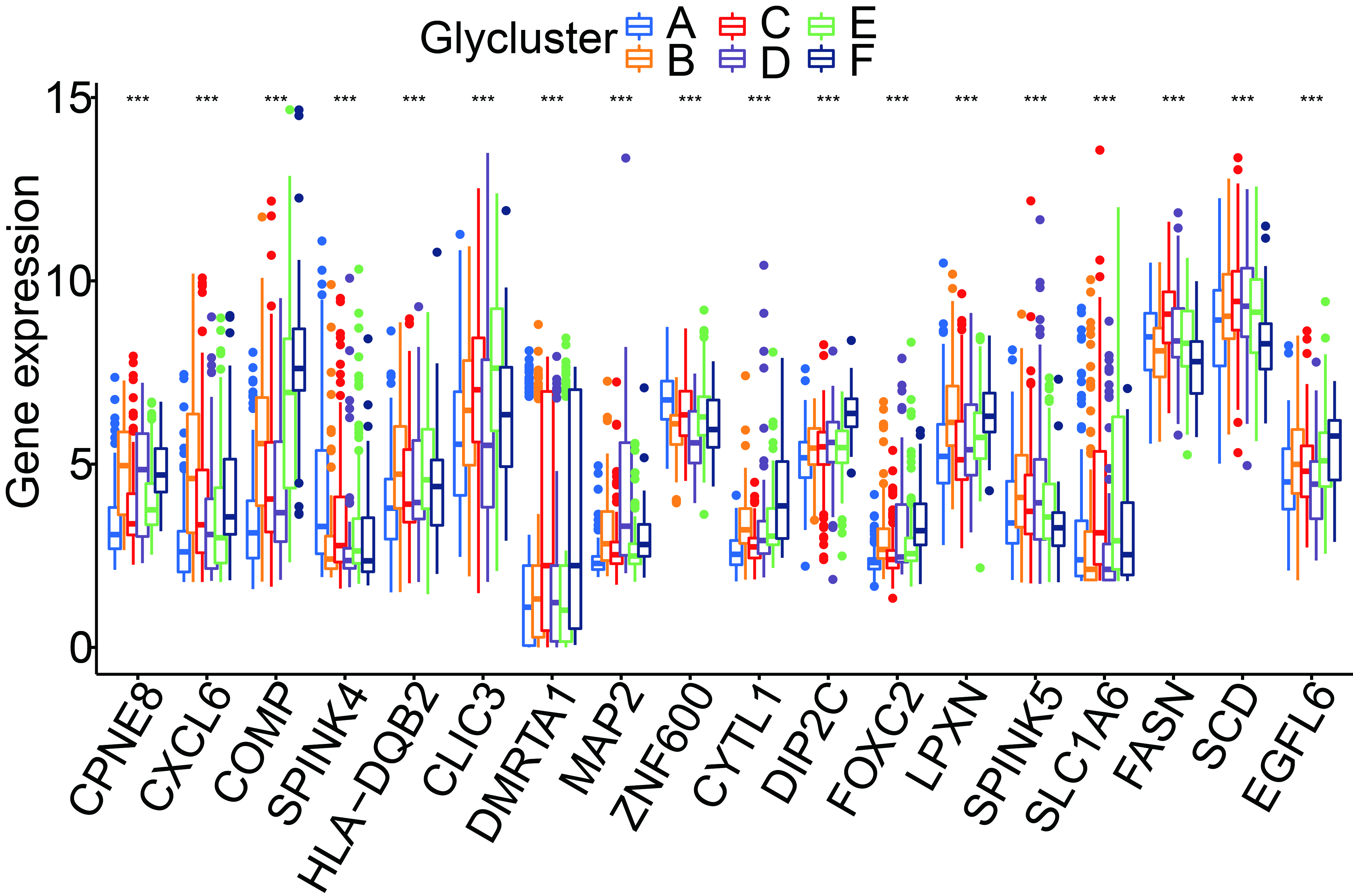

Supplement: Supplementary Figure 8 — The relationship between the expression of the 18 model genes and the glycolysis-related six clusters established by us. [file Image8.tif]

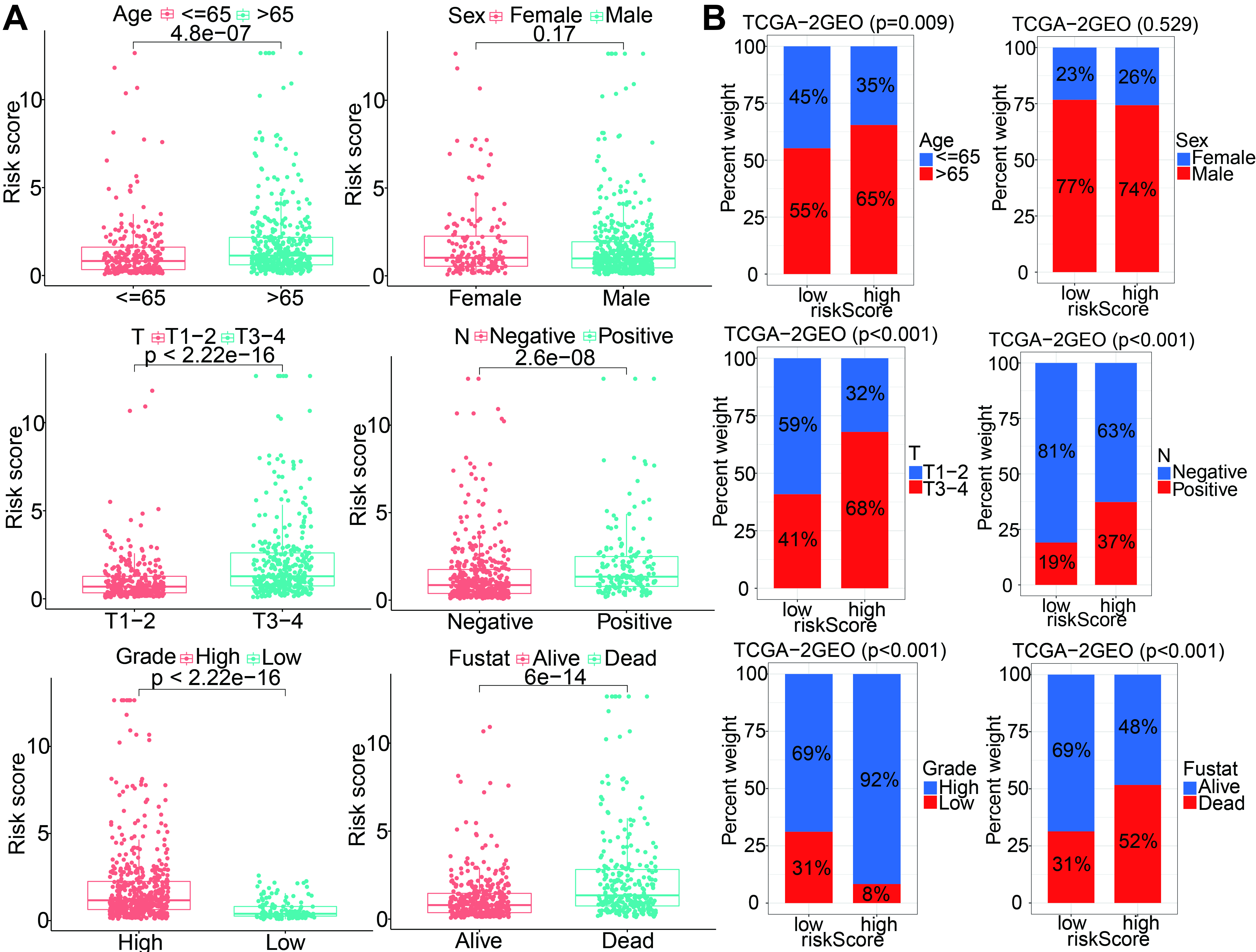

Supplement: Supplementary Figure 9 — Association analysis between risk score/group and clinicopathological data of BCa patients through wilcox rank test or chi-square test based on the merge dateset. [file Image9.tif]

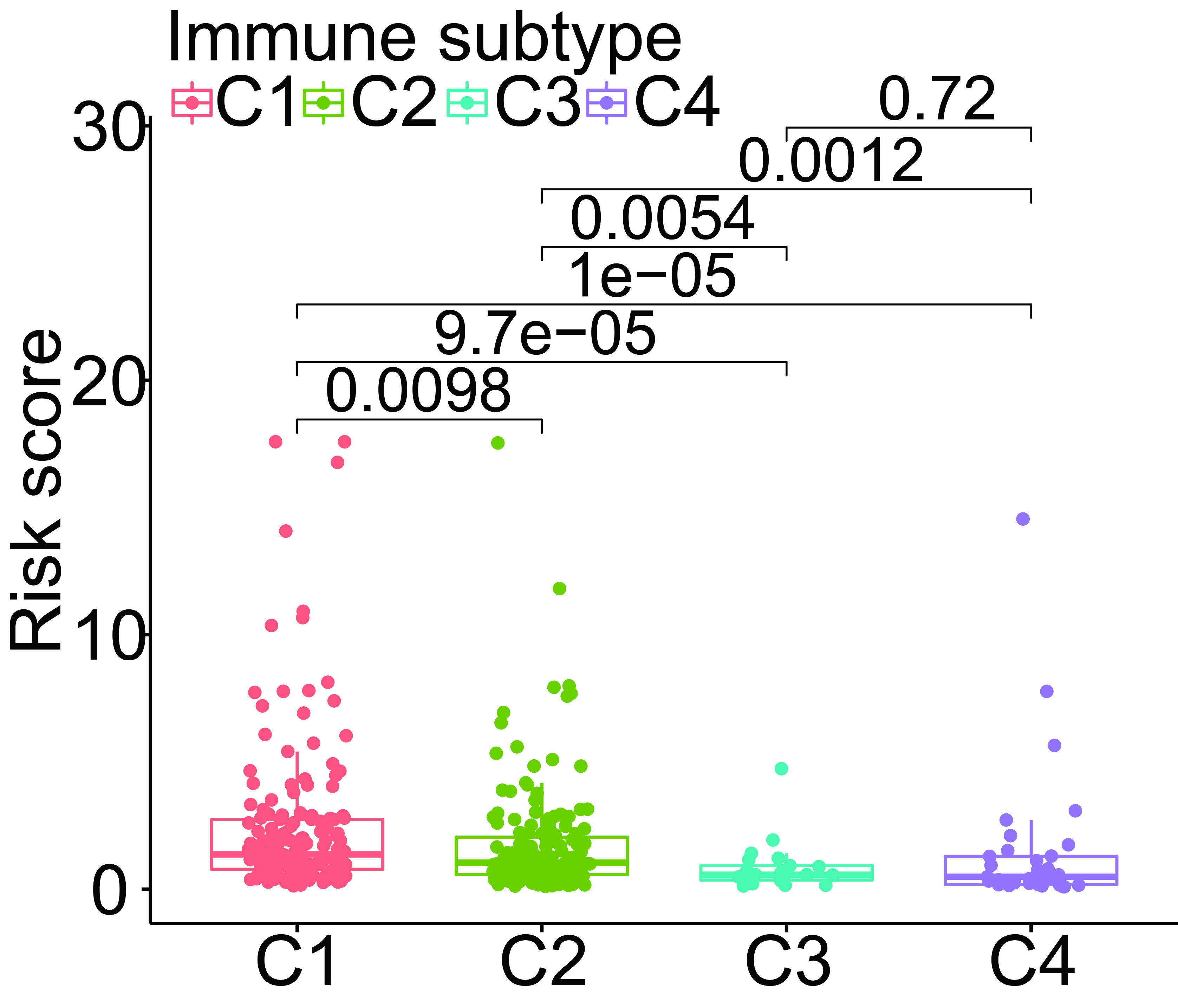

Supplement: Supplementary Figure 10 — Correlation analysis between the modeled risk score and accepted immunotyping results from the TCGA-BCa cohort. [file Image10.tif]

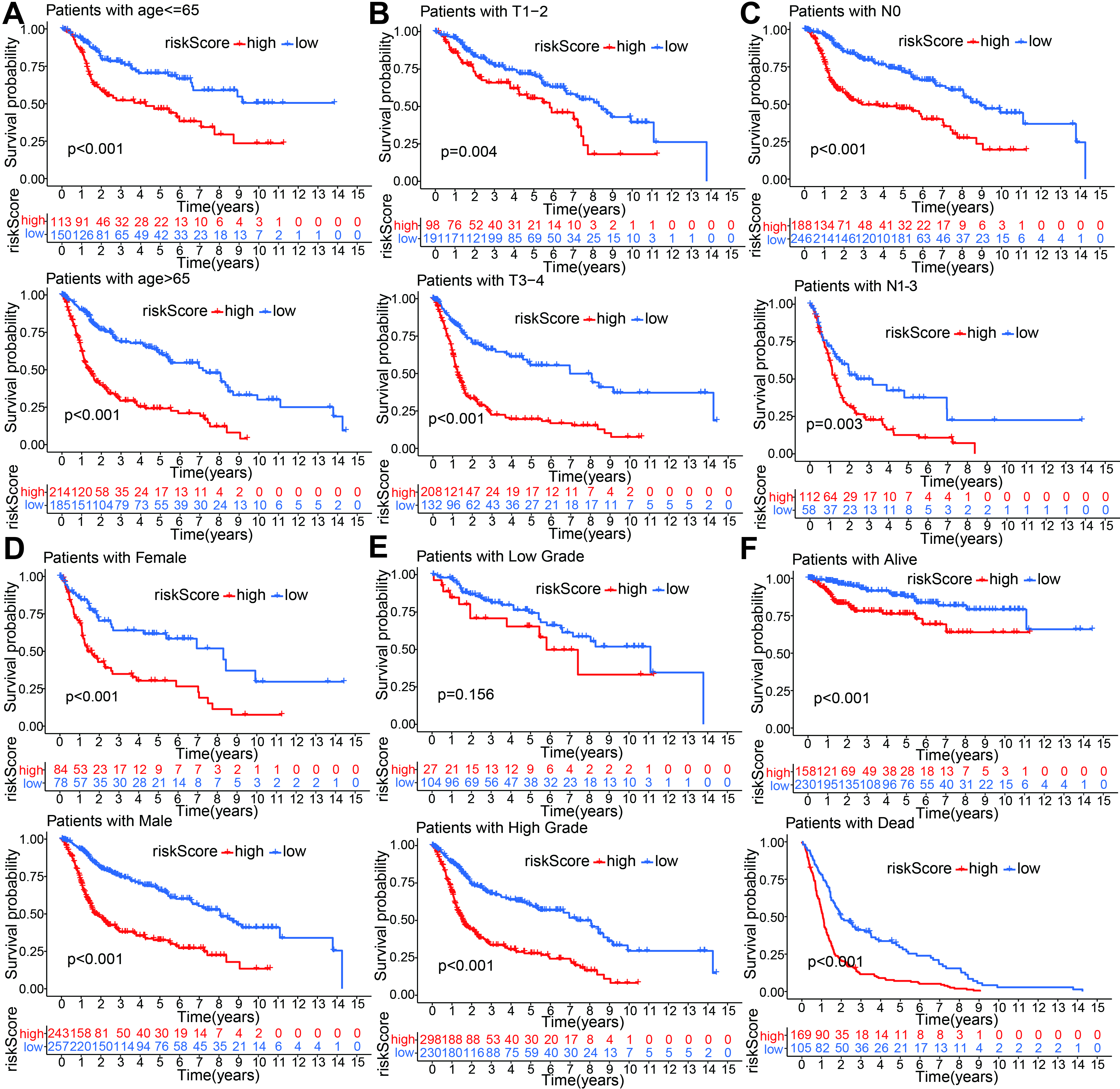

Supplement: Supplementary Figure 11 — Stratification analysis of various clinicopathological factors by Kaplan–Meier curves for the patients with bladder cancer in the high and low risk score groups. Kaplan–Meier curves of OS in different subgroups stratified by (A) age, (B) stageT, (C) stage N, (D) gender, (E) grade, and (F) survival status. [file Image11.tif]

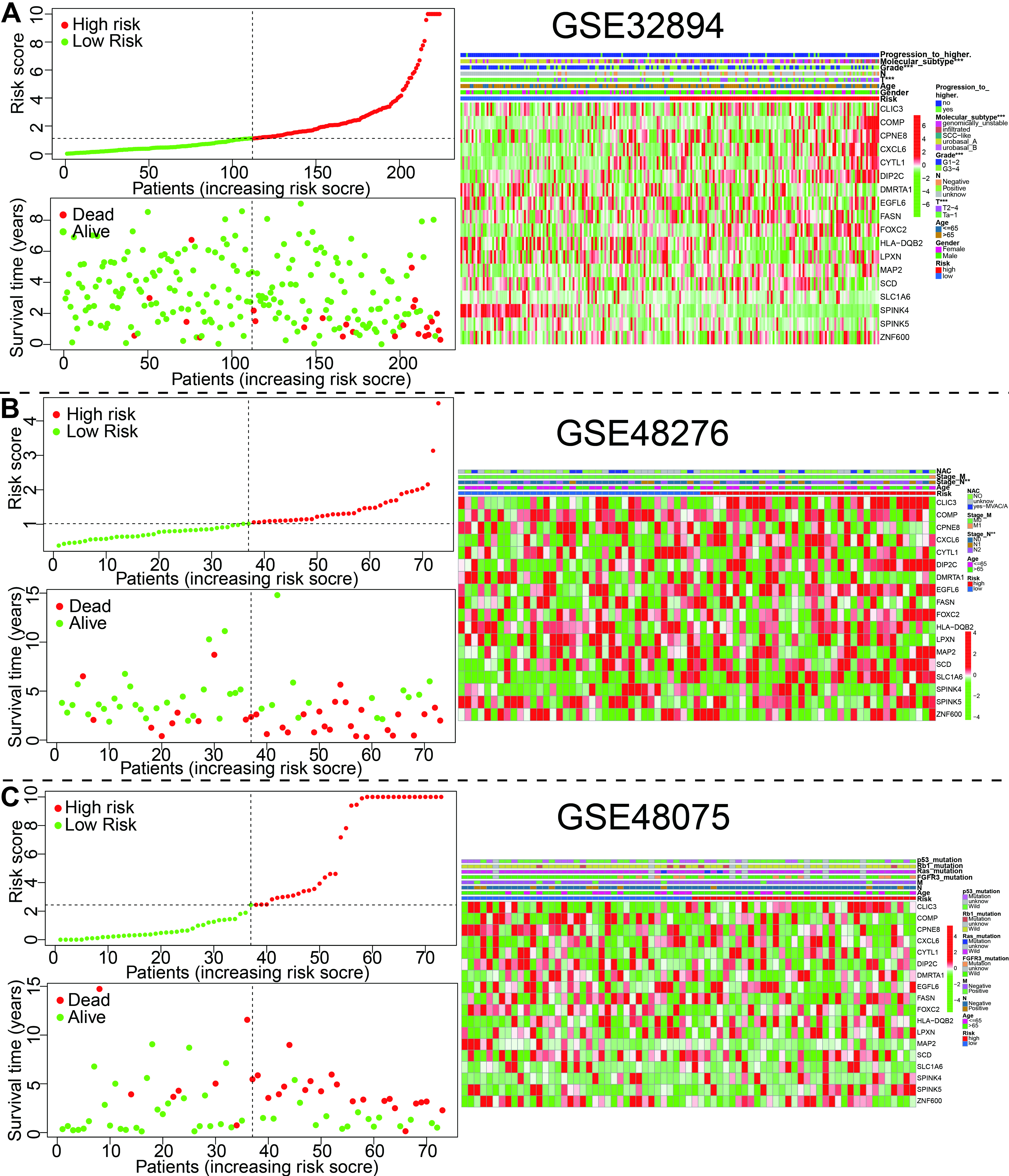

Supplement: Supplementary Figure 12 — The risk score ranking, survival status scatter diagram, and expression heatmap of modeled genes in the (A) GSE32894, (B) GSE48276, and (C) GSE48075 cohorts. [file Image12.tif]

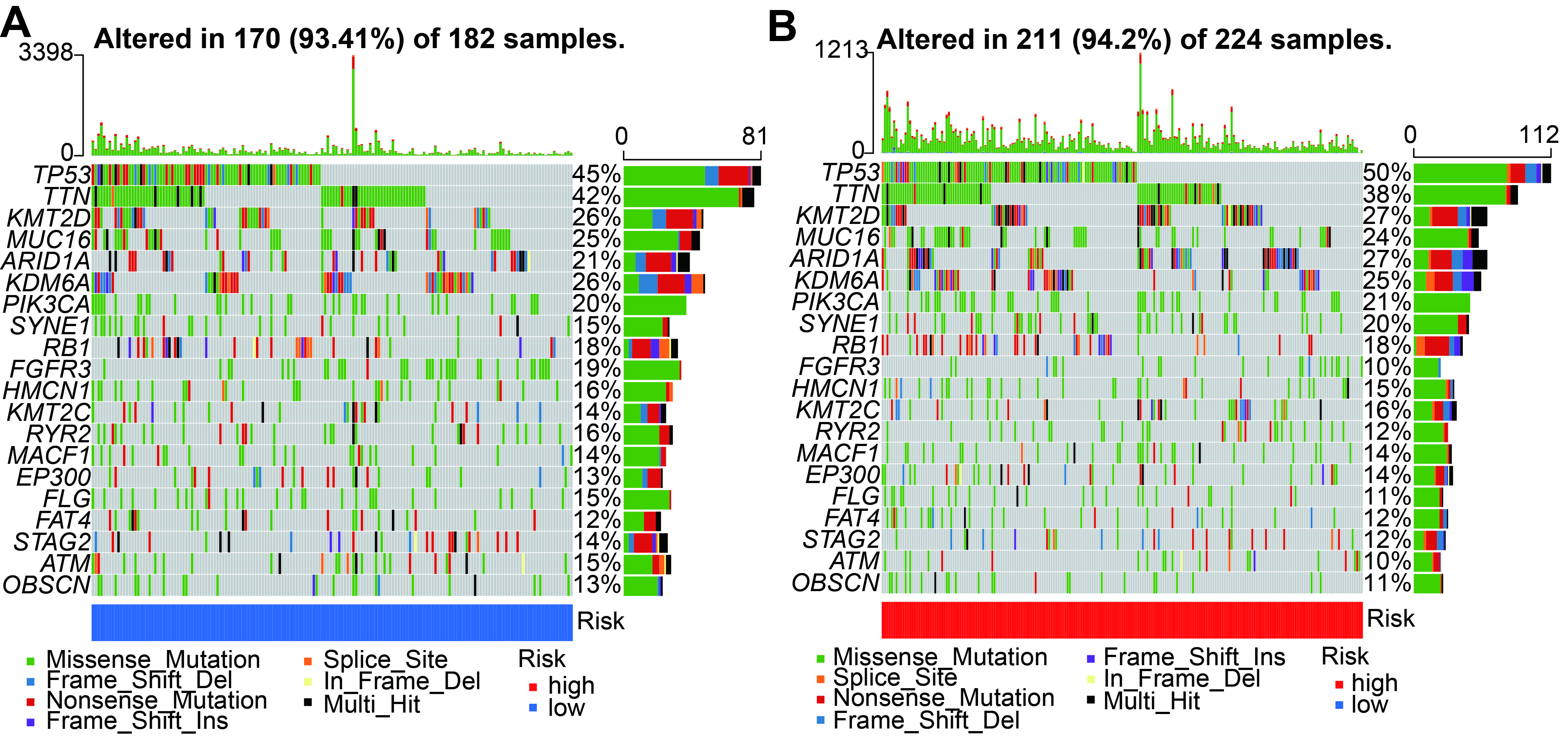

Supplement: Supplementary Figure 13 — The oncoPrint in the low (A) and high (B) risk score groups based on TCGA-BCa cohort. [file Image13.tif]

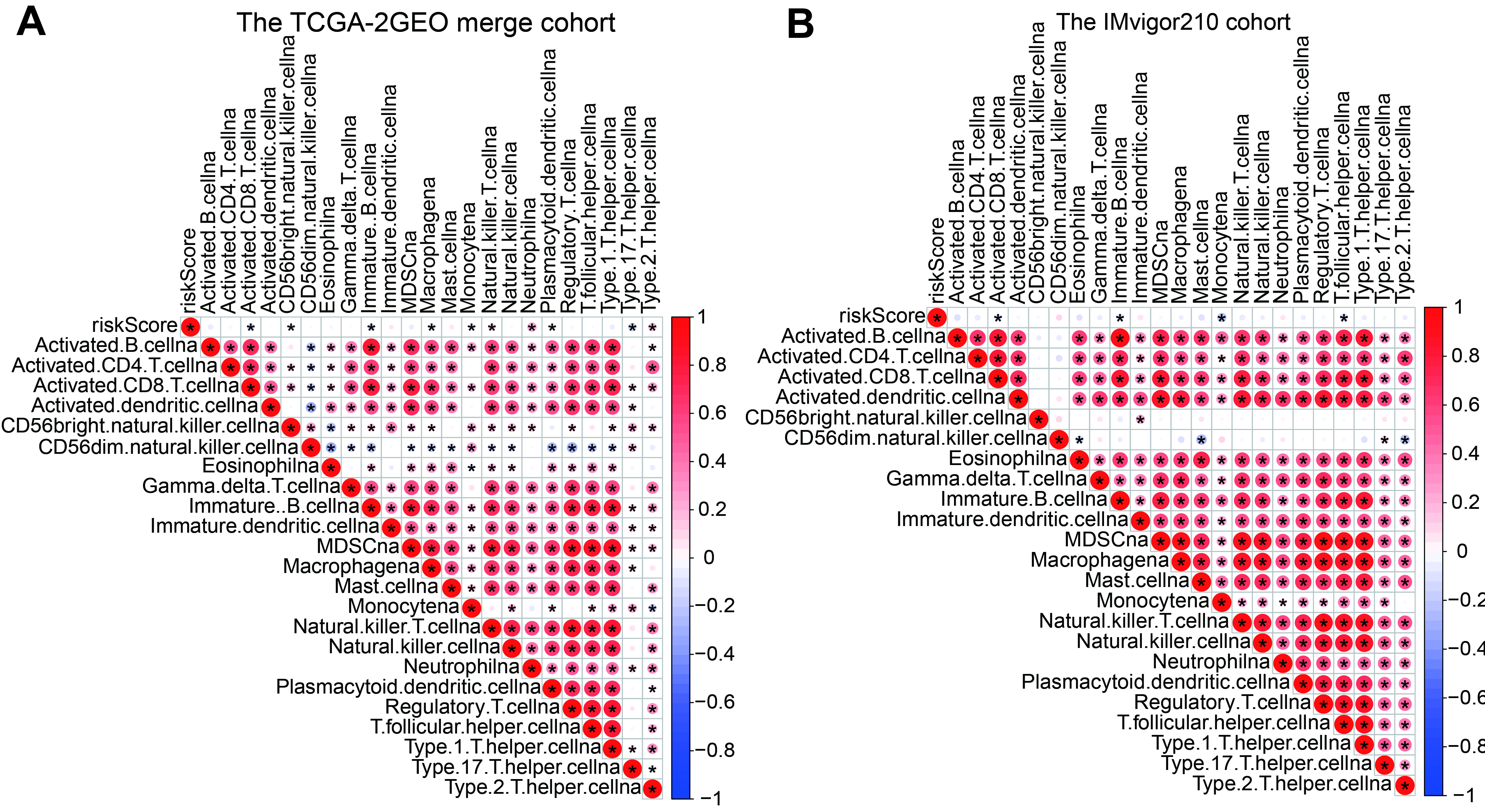

Supplement: Supplementary Figure 14 — Bubble diagram showed the spearman correlation between riskscore and the ssGSEA immune-associated enrichment scores from merge (A) and IMvigor210 (B) cohorts. [file Image14.tif]

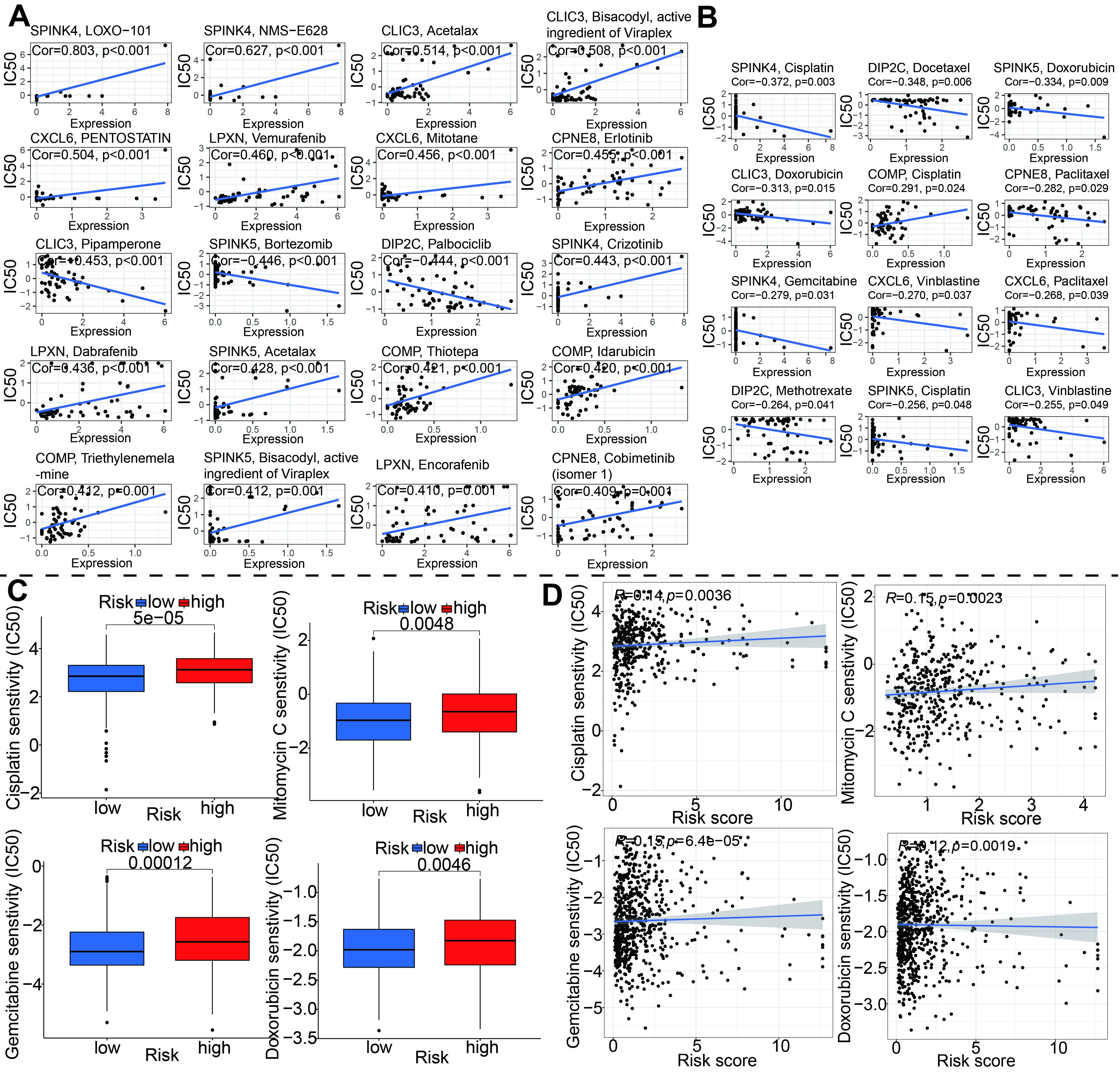

Supplement: Supplementary Figure 15 — Correlations between risk score/group and IC50 for different drugs. (A) The top 20 agents most significantly associated with model gene expression and (B) several clinically commonly used drugs by Spearman rank correlation test with p<0.05 using the CellMiner database. (C) Wilcox group analysis and (D) spearman correlation analysis all indicated that the glycolysis-related gene signature is robust to drug sensitivity of Cisplatin, Gemcitabine, Mitomycin C, and Doxorubicin from the pRRophetic algorithm. [file Image15.tif]
